# Veterinarian barriers to knowledge translation (KT) within the context of swine infectious disease research: an international survey of swine veterinarians

**DOI:** 10.1186/s12917-020-02617-8

**Published:** 2020-11-02

**Authors:** Sheila Keay, Jan M. Sargeant, Annette O’Connor, Robert Friendship, Terri O’Sullivan, Zvonimir Poljak

**Affiliations:** 1 Department of Population Medicine, Ontario Veterinary College, University of Guelph, Guelph, Canada; 2 Centre for Public Health and Zoonoses, Ontario Veterinary College, University of Guelph, Guelph, Canada; 3 Department of Large Animal Clincal Sciences, College of Veterinary Medicine, Michigan State University, East Lansing, Michigan USA

**Keywords:** Knowledge translation, Veterinarian, Swine infectious disease, Survey, Questionnaire, Research synthesis, Evidence based medicine, Information sources

## Abstract

**Background:**

Food animal veterinarians face commodity specific and urgent global challenges yet conditions preventing use of best available knowledge have been sparsely studied. The American Association of Swine Veterinarians (AASV) membership (*N* = 1289) was surveyed online to benchmark their information priorities and their motivations and sources for keeping current with infectious disease research, and to describe their reported time, skill, access, and process as barriers to knowledge translation (KT).

**Results:**

Respondents (*n* = 80) were mostly from Canada (*n* = 40) and the U.S.A (*n* = 31) and demographics approximated the AASV’s. Colleagues are the first choice for information on difficult cases (49%, 95%CI: 38–61). Half of respondents (53%, 95%CI: 41–64) spend an hour or less per week keeping up with infectious disease research. The majority reported moderate or less than moderate efficiency (62%, 95%CI: 51–72), and moderate or greater stress (59%, 95%CI: 48–70) with their process for keeping up. Journal article methods sections are commonly not read, almost a third (32%, 95% CI: 22–43) reported either they do not evaluate statistical methods or that they had poor confidence to do so, and half (52, 95%CI: 41–63) could not explain ‘confounding bias’. Approximately half (55%, 95%CI: 41-69) with direct oversight of swine herds had full access to 2 or fewer academic journals. Approximately a third of respondents (34%, 95%CI: 24–46) selected only formats involving single research studies (either full text or summaries) as preferred reading materials for keeping current over expert summaries of the body of evidence.

**Conclusion:**

KT barriers are considerable and a source of stress for many swine veterinarians. Sub-optimal efficiency with keeping up and low confidence to appraise aspects of research are concerns. Results are consistent with previous literature and illustrate need for improved KT infrastructure and for additional training in statistical methods and interpretation of primary research. Further evaluation is warranted of why approximately a third of veterinarians in this study, for the purpose of keeping up, preferentially choose to review individual research studies over choices that would include an expert summary of the body of evidence. Consideration of reasons for this preference will be important in the planning of KT infrastructure improvements.

**Supplementary information:**

**Supplementary information** accompanies this paper at 10.1186/s12917-020-02617-8.

## Background

### Evidence based medicine (EBM), knowledge translation (KT) and barriers to KT

Within health professions, evidence based medicine (EBM) was described by Sackett [1] as the integration of individual clinical expertise with the best available external body of evidence. Within an EBM framework, putting knowledge to use is referred to as knowledge translation (KT) [2]. Both tacit and explicit knowledge are needed for the practice of EBM [1]. Explicit knowledge relates to methodological rigor whereas tacit knowledge is related to “relevance or real-world viability” [3, 4]. For the purposes of this report, peer-reviewed primary research literature was considered exclusively as explicit knowledge.

Knowledge translation (KT) includes the iterative processes of synthesis, dissemination, exchange, and application of knowledge [5]. Barriers, or the factors which impede knowledge translation, vary in type and impact depending on the type of knowledge [4], the directional flow of information, and the population level or levels being investigated [6, 7]. Time, access, and skill, identified as individual level KT barriers [8–10], have been sparsely studied in veterinary medicine [11–13]. We consider also process as a fourth barrier, or the sequential steps taken from wanting current research information through to its assimilation into practice.

### Veterinarian KT surveys

Veterinary access to information has significantly changed since the 1990’s. Surveys exploring veterinarian KT barriers or continuing education and continuing professional development (CE/CPD) prior to this time may therefore have limited applicability. Additionally, species specialization influences CE/CPD learning priorities. The role of food animal and agri-food public health veterinarians includes also protection of the quality, security, and sustainability of food for human consumption [14, 15], and they prefer topics of preventive and population medicine [16–18]. As such, modern applied food animal research is often population focused, publicly funded, and global in applicability, making it distinct from other areas of veterinary research [19–22].

### Swine veterinarians and KT

Swine veterinarians are a specialized, progressive, and cohesive community of practice [23]. On-going emergence and re-emergence of swine diseases of economic and potentially public health significance [24, 25], have made for heightened, dynamic, and sometimes urgent KT needs [26]. Three published surveys focus specifically on swine veterinarians; Penny and Penny [27] (1978) identified pig research priorities and summarized gaps between swine veterinarian interests and available research, a Dutch language survey by Maes (2010) [28], focused on Belgian demographics and practice functioning, and a 2010 survey of Ontario swine industry stakeholders, inclusive of swine veterinarians, showed veterinarians had a preference for producer meetings as a source of educational material [29].

### Survey objectives

The survey objectives were to benchmark swine veterinarian overall priority interests, their motivations for seeking, and their sources used to find research information, and to describe how time, process, access, and skill to understand and to assess research (hereafter referred to as skill), may act as barriers to keeping current with infectious disease research.

## Results

### Survey respondent metrics

Ninety-four respondents opened the online link, 10 exited at the consent page, 1 answered only first block questions, 2 identified as non-veterinarians, and 1 omitted to identify role, leaving 80 usable responses. Response rate was just over 6% (83/1289) of AASV membership; however, approximately 29% of the CASV membership participated (M. DeGroot, personal communication).

### Demographics

**Fig. 1:**
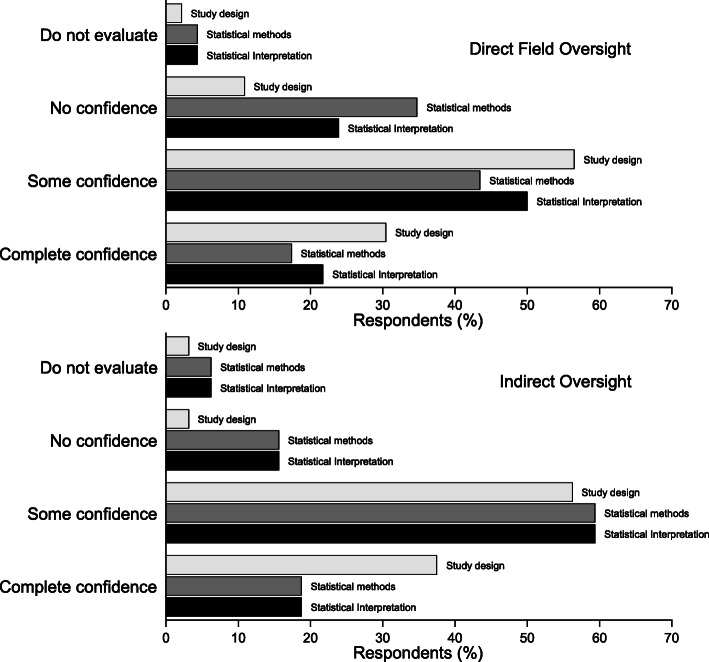
(Q12) Self-reported confidence to evaluate a research paper for the appropriateness of the study design used, the statistical methods used, and the author’s statistical interpretation of results, by role (direct vs. indirect) (Data supporting this figure are shown in Table 5 in Additional file 3)

Respondent demographics are detailed in Table 1 in Additional file 3. By veterinary role, composition closely matched that of the AASV membership (see Fig. 1 in Additional file 3). Overall, most respondents worked either in Canada (50%, *n* = 40) or the U.S.A. (39%, *n* = 31), most were experienced, with two thirds (64%, *n* = 51) reporting more than 15 years of working with swine, and half (51%, *n* = 41) working exclusively with swine. Eleven percent (*n* = 9) provided direct oversight to greater than 100,000 sows worth of production each (100,000 sows translates into approximately 2 million market offspring/year). Two demographic criteria were used to dichotomize respondent’s role as ‘direct’ (*n* = 47) or ‘indirect’ (*n* = 32) (See Table 2A and B in Additional file 3). Most of the respondents designated as ‘indirect’ also reported zero (0) sows worth of production under their veterinary care (86%, *n* = 28) (See Table 2D in Additional file 3).

### Swine veterinarian priority interests

Respondents most sought information on disease control, emerging diseases, and disease control interventions, inclusive of globally endemic production viruses (PRRS, IAV-S, PED), and bacteria (*M. hyopneumoniae, L. intracellularis, S. suis*) (Table 1). Veterinarians with indirect oversight were almost 4 times as likely to select antimicrobial resistance as one of their top three interests compared with veterinarians with direct oversight (prevalence ratio (PR)= 3.9, 95%CI: 1.1–13.7, *p* = 0.04). Those with direct field oversight were 3 times as likely to select antimicrobials (PR= 2.9, 95%CI: 1.2–6.8, *p* = 0.008) as one of their top three interests, 3 times as likely to include *Haemophilus parasuis* amongst their top three swine bacteria of interest (PR = 2.9, 95%CI: 1.07–7.80, *p* = 0.02), and also more likely to select IAV-S as a priority virus (PR = 1.6, 95%CI: 1.08–2.30, *p* = 0.01) versus veterinarians with indirect oversight.

**Table 1:**
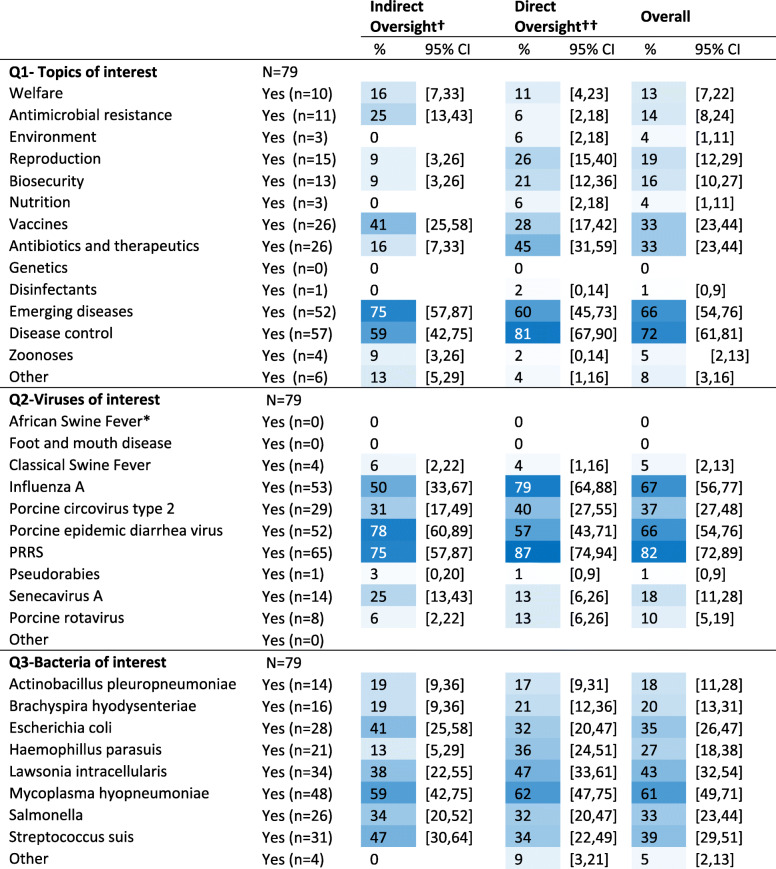
Respondent selections, by veterinary role, of general topics (Q1), swine viruses (Q2), and swine bacteria (Q3), for which information is most often sought ^Ŧ^. Respondents could select up to three [3] choices per question

† Indirect oversight = non-practitioner that provides 1000 sows worth or less with direct veterinary services

†† Direct oversight = practitioner or a non-practitioner that provides > 1000 sows worth with direct veterinary services

* Note, African Swine Fever had not yet been diagnosed in Asia at the time of this survey

Ŧ Cells are color conditioned on blue to white with higher percentages as darkest blue

### Motivation to seek infectious disease information

Difficult cases most motivated veterinarians with direct oversight to seek information (Table 2) and were three times as likely to be a motivator for direct versus indirect veterinarians (PR = 3.1, 95%CI: 1.4–6.6, *p* < 0.01). Veterinarians with indirect oversight were over 6 times as likely to be motivated by other work requirements versus direct veterinarians (PR = 6.2, 95%CI: 2.3–16.8, *p* < 0.01).

**Table 2:**
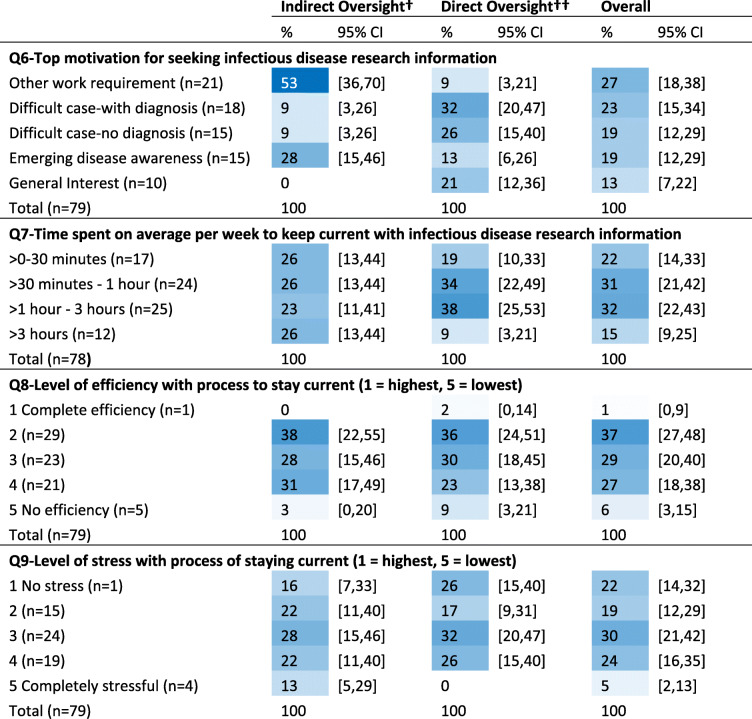
Frequency ^Ŧ^ of self-reported top motivation for seeking information(Q6) average time spent per week (Q7), process efficiency (Q8), and level of stress (Q9) with staying current with swine infectious disease research, by veterinary role

† Indirect oversight = non-practitioner that provides 1000 sows worth or less with direct veterinary services

†† Direct oversight = practitioner or a non-practitioner that provides > 1000 sows worth with direct veterinary services

Ŧ Cells are color conditioned on blue to white with higher percentages as darkest blue

### Barriers to knowledge translation

#### Time

About half of all respondents (53%, 95% CI: 41–64) spend 1 h or less per week keeping current or reviewing infectious disease research information (Table 2). Veterinarians with indirect oversight were 3 times as likely as those with direct oversight to spend greater than 3 h per week at this task (PR = 3.0, 95%CI: 1.0–9.2, *p* = 0.05).

#### Process

Respondents described, on a scale from 1 to 5, their level of efficiency and their level of stress with the process of keeping up with infectious disease research. More than half of all respondents rated their level of stress and their level of efficiency, at best, as moderate (Table 2). When outcomes were collapsed into dichotomous responses respondents reporting ‘high to complete efficiency’ versus ‘moderate to no efficiency’, were 1.8 times more likely to also report spending greater than an hour per week keeping current (PR = 1.8, 95%CI: 1.2–2.9, *p* = 0.02), and more likely to report having confidence to assess statistical methods (PR = 1.4, 95%CI: 1.1–1.9, *p* = 0.03). Stress and efficiency were reciprocally associated. Respondents reporting the lowest levels of stress were more than twice as likely to also report the highest levels of efficiency (PR = 2.2, 95%CI:1.3–4.0, *p* = 0.01). Those motivated by difficult cases to seek information were more than twice as likely to also report the highest levels of stress (PR = 2.2, 95%CI 1.1–4.5, *p* = 0.04). Respondent reported levels of stress and efficiency did not differ by role (see Table 3 in Additional file 3).

#### Skill

##### Familiarity with knowledge terms

Respondents were asked to describe their level of understanding of each knowledge term as ‘can explain’, ‘have heard of’, or as ‘not familiar with’ (see Table 4 in Additional file 3). Consistency of self-reported levels of understanding, as measured by Cronbach’s alpha, was poor for the grouping of vaccine terms (efficacy, effectiveness, and basic reproductive number) (Cronbach’s alpha = 0.54) and for the grouping of EBM terms (EBM, EBVM, and evidence pyramid) (Cronbach’s alpha = 0.69), but was strong for the grouping of bias terms (information, selection, and confounding bias) (Cronbach’s alpha = 0.91). Veterinarians with indirect oversight were twice as likely versus veterinarians with direct oversight to report not having heard of the term evidence pyramid (PR = 2.2, 95%CI: 1.2–4.1, *p* = 0.02). When responses were dichotomized as either ‘can explain’ or as ‘cannot explain’ (Table 3), ability to explain was strongest for the terms vaccine efficacy and vaccine effectiveness, and poorest for the terms evidence pyramid, basic reproductive number, and confounding and information biases.

**Table 3:**
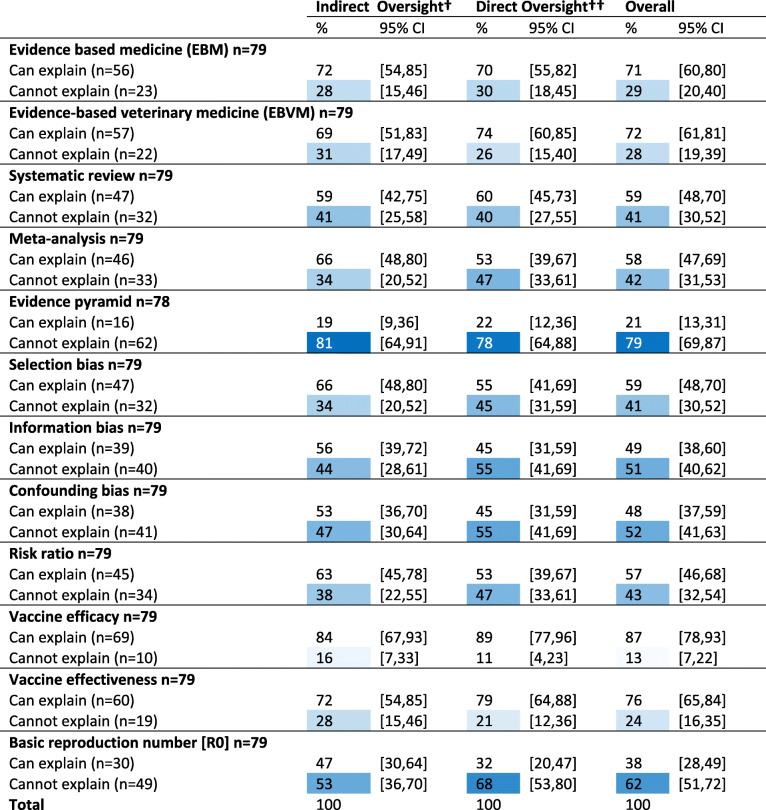
(Q10) Frequency ^Ŧ^ of self-reported level of familiarity with epidemiologic and EBM terminology with outcomes dichotomized* as ‘can explain’ or ‘cannot explain’, by veterinary role

* See Table 2 in Additional file 3 for details on how categories were collapsed into dichotomous options

† Indirect oversight = non-practitioner that provides 1000 sows worth or less with direct veterinary services

†† Direct oversight = practitioner or a non-practitioner that provides > 1000 sows worth with direct veterinary services

Ŧ Cells with % responses for “Cannot explain” are color conditioned on blue to white with higher percentages as darkest blue

##### Confidence to assess technical aspects of a research study

Respondents were asked to describe their level of confidence to evaluate the appropriateness of study designs used, the statistical methods used, and study author’s statistical interpretation of findings. Internal consistency of responses was good (Cronbach’s alpha = 0.89) and the majority of respondents, regardless of role, reported having ‘some confidence’ to evaluate each of the three aspects of a study (Fig. 1).

When response options were dichotomously aggregated approximately a quarter (26%, 95%CI: 17–37) of respondents reported ‘no confidence to evaluate/do not evaluate’ the statistical interpretation of findings, and almost a third (32%, 95%CI: 22–43) reported ‘no confidence to evaluate/do not evaluate’ the statistical methods used (see Table 6A in Additional file 3). Direct veterinarians were almost twice as likely versus indirect veterinarians to report ‘no confidence to evaluate/do not evaluate’ the statistical methods used (PR = 1.8, 95%CI: 1.1–2.8, *p* = 0.01). Confidence to evaluate technical aspects of studies was not significantly associated with frequency of reading methods sections of articles (See Table 6B in Additional file 3).

Consistency in the reported confidence to assess the technical aspects of a study with reported levels of understanding of each of the terms ‘confounding bias’, ‘information bias’, and ‘selection bias’ was good (Cronbach’s alpha = 0.84, 0.84, and 0.83 respectively). Respondents who reported being unable to explain confounding bias, versus those who could, were 2.8 times more likely to also report ‘no confidence/do not assess’ the statistical interpretation of study findings (PR = 2.78, 95%CI: 1.1–6.9, *p* = 0.02).

#### Access

##### Ranking of 1st, 2nd and 3rd choices for information on difficult cases

**Fig. 2:**
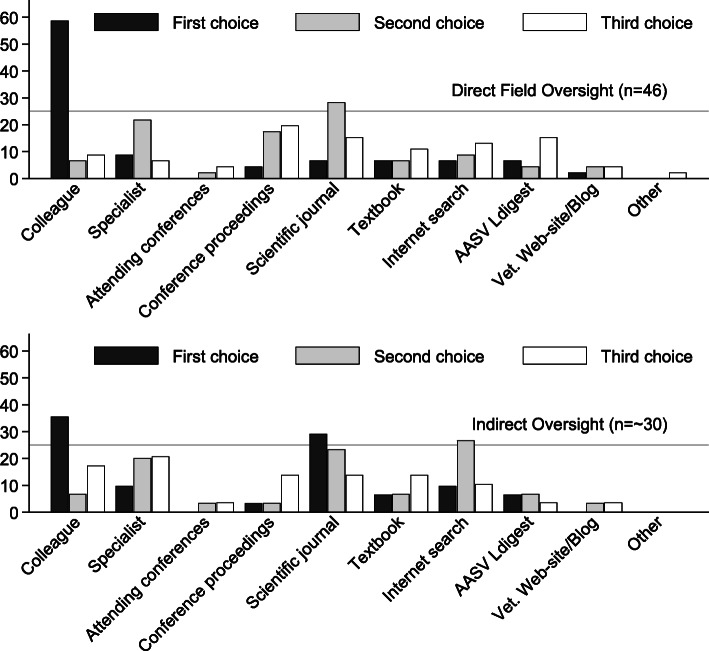
(Q4) Frequency (%) of rankings of first, second, and third choices for getting information for difficult clinical cases by role (direct vs indirect) (data supporting this figure are shown in Table 7 in Additional file 3)

Overall, colleagues were the most frequently selected first choice for seeking information on difficult cases (49%, 95%CI: 38–61), scientific journals as the second choice (26%, 95%CI: 17–38), and conference proceedings as the third choice (17%, 95%CI: 10–28) (Fig. 2). The respondents’ ordering of rankings was varied and included 60 different combinations of the three choices with colleague-specialist-journals being the most frequent ordering (5%).Veterinarians with direct oversight were 1.7 times more likely (95%CI: 1.0–2.9, *p* = 0.04) to select colleagues as a first choice, and were 2.1 times as likely to have picked proceedings as one of a 1st, 2nd, or 3rd choice (95%CI: 1.0–4.8, *p* = 0.05), versus indirect veterinarians, whereas indirect veterinarians were 4.5 times more likely to select scientific journals as a first choice (95%CI: 1.3–15.1, *p* = 0.01) versus direct veterinarians.

##### Journal reading habits

**Fig. 3:**
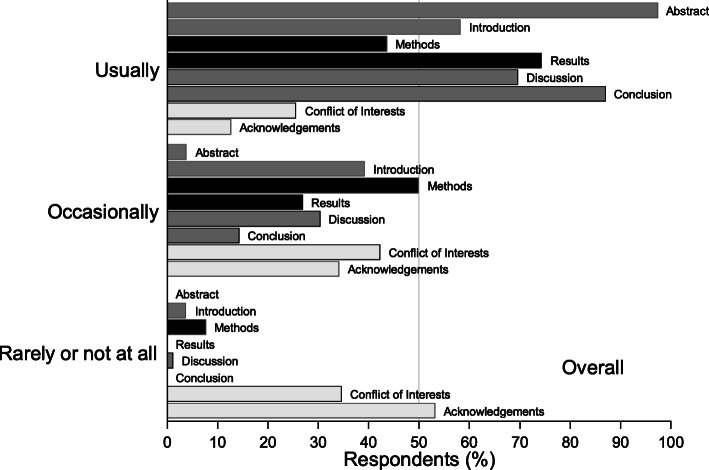
(Q11) Overall reported frequency (%) of reading of sections of journal articles (*n* = 79) (data supporting this figure are shown in Table 8 in Additional file 3)

The most frequently read sections of scientific journal articles were the abstract (97%, 95%CI: 90–99) and conclusions (86%, 95%CI: 76–92), followed by results (73%, 95%CI: 62–82) and the discussion (7%0, 95%CI: 58–79) (Fig. 3). Less than half of respondents usually read the methods (42%, 95%CI: 32–54). There were no significant differences by role in the frequency of ‘usually read’ versus ‘not usually read’ sections.

##### Use of search methods

Respondents were asked to select their two most preferred methods to search for swine infectious disease research information from a list of 6 options. Online options were preferred with the online AASV Information Library (61%, 95%CI: 49–71), followed by Google or Google Scholar (52%, 95%CI: 41–63), and bibliographic databases (51%, 95%CI: 40–62) as most frequently selected (Table 4). The AASV library, available online to AASV members only, provides access to conference proceedings from 8 national and international swine meetings, to the Journal of Swine Health and Production (JSHAP), and to a PDF of the textbook *Diseases of Swine*. Seventy-four percent of veterinarians with direct field oversight (95%CI: 60–85) most preferred the AASV library and were 1.8 times more likely to do so over veterinarians with indirect oversight (PR = 1.83, 95%CI: 1.16–2.89, *p* = 0.004). There were no other significant differences by role in choice of preferred methods for searching for research information.

**Table 4:**
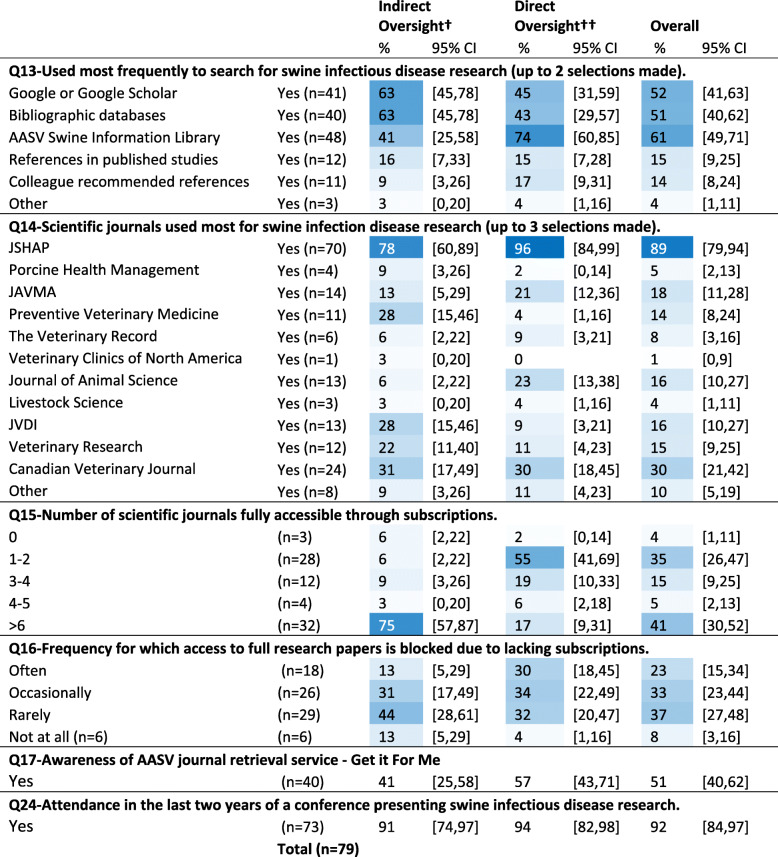
Selection frequencies (%) ^Ŧ^ of most used search methods (up to two [2] options) (Q13) most used scientific journals for infectious disease information (up to three [3] options) (Q14), number of journals with subscription access (Q15), frequency of blocked full text access (Q16), awareness of article retrieval service (Q17), and conference attendance (Q24), by role

† Indirect oversight = non-practitioner that provides 1000 sows worth or less with direct veterinary services

†† Direct oversight = practitioner or a non-practitioner that provides > 1000 sows worth with direct veterinary services. Ŧ Cells are color conditioned on blue to white with higher percentages as darkest blue

JSHAP = Journal of Swine Health and Production, JAVMA = Journal of the American Veterinary Medical Association, JVDI = Journal of Veterinary Diagnostic Investigation

##### Use of journals

Respondents were asked to identify up to three journals they used most for swine infectious disease information. The list of provided options captured all but 10% (95%CI: 5–19) of the respondent selections (Table 4). The AASV publication JSHAP was selected most (89%, 95%CI: 79–94), followed by the Canadian (30%, 95%CI: 21–42), and the American (18%, 95%CI: 11–28) Veterinary Association Journals, each of which is freely available with membership. Forty-one percent (95%CI: 30–52) reported access to greater than 6 scientific journals versus 35% (95%CI: 41–69) reporting access to 1–2 journals (Table 4). Direct veterinarians were almost 9 times more likely to report access to 1–2 journals versus indirect veterinarians (PR = 8.9, 95%CI: 2.3–34.7, *p* < 0.01), and, indirect veterinarians were over 4 times more likely to report access to > 6 journals (PR = 4.4, 95%CI: 2.3–8.5, *p* < 0.01.

Overall, 37% (95%CI: 27–48) of respondents reported online full journal article access was rarely blocked, 33% (95%CI: 23–44) as occasionally blocked, and 23% (95%CI: 15–34) as often blocked (Table 4). There were no significant differences by role in reported blocked access. Of respondents reporting access to 1–2 journals only, almost half (48%, 95%CI: 30–67) reported occasionally being blocked, and 39% (95% CI: 19–63) as often blocked (see Table 9 in Additional file 3). Over half of respondents reporting rarely being blocked, also reported access to > 6 journal subscriptions (PR = 55, 95%CI: 37–72), but interestingly almost a third (31%, 95%CI: 17–50) of those reporting rarely being blocked also reported having access to 1–2 journals only (see Table 9 in Additional file 3). Overall, half (51%, 95%CI: 40–62) of respondents were aware of the Get-It-For-Me free membership service (Table 4) including 61% (95%CI: 37–81) of those also reporting online journal article access as often blocked (see Table 9 in Additional file 3). In the last two years, almost all respondents had attended a conference where swine infectious disease research information was presented (Table 4).

### Preferred reading material format

Respondents were asked to select from a list of 6 options their two [2] preferred formats for reading materials for keeping current on infectious disease topics (Table 5). Original individual primary research papers (IPRP) and one-page expert critical summaries of an IPRP were the most frequently selected options (both selected at 47%, 95%CI: 36–58) and full narrative expert critical review of the body of evidence (BOE) was least selected (15%, 95%CI:9–25). Preferences were not significantly different by role (see Table 10 in Additional file 3).

**Table 5:**
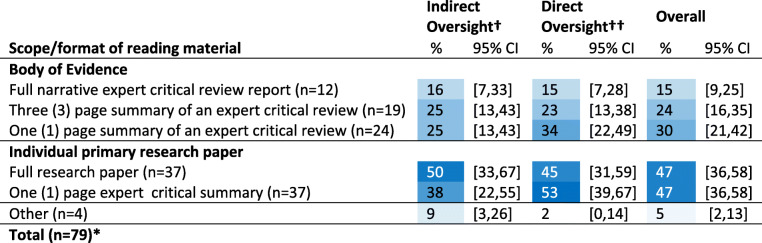
(Q18) Selection (%) ^Ŧ^ of preferred reading material formats for keeping current with infectious disease research (given a choice of up to two [2] options), by role

† Indirect oversight = non-practitioner that provides 1000 sows worth or less with direct veterinary services

†† Direct oversight = practitioner or a non-practitioner that provides > 1000 sows worth with direct veterinary services

Ŧ Cells are color conditioned on blue to white with higher percentages as darkest blue

*Total does not add to 79 as respondents chose up to 2 options, **Other options reported included podcasts to listen to while driving, abstracts from papers, a summary report of the evidence, and a systematic review

**Fig. 4:**
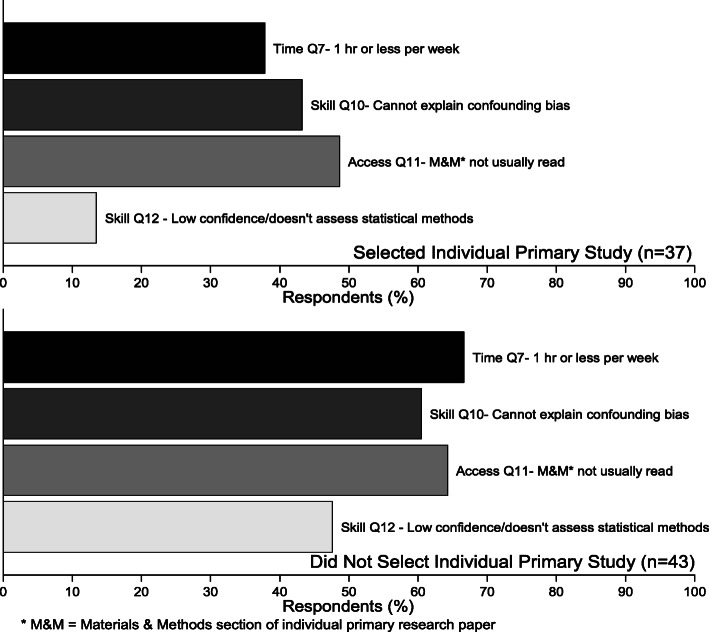
(Q18) Comparison of frequencies (%) of respondents reporting all of four defining KT barriers between those who selected for keeping current full text individual primary research papers (IPRP) versus those who did not. Defining KT barriers include 1) an hour or less available per week to keeping up, 2) low confidence to assess statistical methods used in a study, 3) inability to explain confounding bias, and 4) does not usually read methods section) (data supporting this figure are shown in Table 11A in Additional file 3)

We compared respondents’ preference for the full IPRP against their responses to questions on aspects of time, skill, and access (Fig. 4). Of respondents selecting a full IPRP as a preferred option, 43% (95%CI: 28–60) also reported not being able to explain the term confounding bias, approximately half (49%, 95%CI: 33–65) did not usually read the materials & methods section of articles, and 14% (95%CI: 6–29) reported they have no confidence to assess, or that they do not assess, the appropriateness of the statistical methods used in an IPRP. Respondents who did not select full IPRPs were almost twice as likely to spend an hour or less per week keeping current (PR = 1.8, 95%CI: 1.1–2.8, *p* = 0.01) versus those who selected full IPRPs, and 3.5 times more likely to also report they have no confidence or do not evaluate the statistical methods used in a research study (95%CI: 1.5–8.5, *p* < 0.01) (see Table 11B in Additional file 3).

The most frequently selected combination of options included both a format involving an expert summary of the body of evidence (BOE) and a format involving content from an individual primary research paper (IPRP) (42%, 95%CI: 31–53) (Table 6). Over a third of respondents (34%, 95%CI: 24–46) selected exclusively options involving IPRPs. There were no significant differences in the selection of combinations by veterinarian role (see Table 12 in Additional file 3). Respondents reporting ‘moderate to high stress’ were 4.3 times more likely to select exclusively BOE options versus respondents reporting ‘little to no stress’ (PR = 4.3, 95%CI: 1.0–17.9, *p* = 0.02).

**Table 6:**
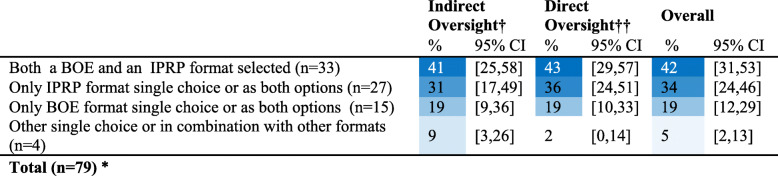
(Q18) Frequency (%)^Ŧ^ of selected two-option combinations of preferred content type (BOE or IPRP) for reading materials‡ used to keep current with research on a specific disease topic, by role

Ŧ Cells are color conditioned on blue to white with higher percentages as darkest blue

BOE = content summarizing body of evidence, IPRP = content derived from a single individual primary research paper

‡ reading material formats could be either a full report, a 3 page summary, or 1 page summary

† Indirect oversight = non-practitioner that provides 1000 sows worth or less with direct veterinary services

†† Direct oversight = practitioner or a non-practitioner that provides > 1000 sows worth with direct veterinary services

*Total does not add to 79 - respondents chose up to 2 options

## Discussion

Veterinary licensing bodies expect licenced veterinarians to keep aware of the significance, relevance and validity of current research [30]. Continuing education (CE) and continuing professional development (CPD) are often mandatory obligations for licensure and are, for the most part, self-directed by veterinarians [30]. Cumulative increases in research technical rigor suggests however, that staying current is an increasingly onerous individual burden [31–35].

### Time

Time is the most consistently reported KT barrier across all previous veterinarian surveys and a consistently reported obstacle to participation in CE/CPD [10, 12, 16, 36–43]. Understandably then, time constrained swine veterinarians seek colleagues, may select citations by convenience [12, 44], or seek narrative reviews for keeping up; with the latter two being at odds with the practice of EBM [45–49]. Synthesis has been described as the basic unit of knowledge translation KT [11] and within an EBM framework research synthesis involves robust and consistent steps to mitigate risk of bias when assimilating knowledge [50]. The impact of how veterinarians allocate time on their effectiveness with keeping up will be important to evaluate further. For example, over 275 primary research publications were identified as relevant following a recent search for primary literature on IAV-S vaccines in swine [51]. It may not be a pragmatic choice for many of the respondents in this survey to try to access and to assess this body of evidence simply given the reported time they allot each week to staying current.

### Process efficiency and stress

Veterinary professional stress has been reported elsewhere [52]. The substantial proportion of swine veterinarians reporting high levels of stress and limited efficiency with the process of keeping up is concerning. Veterinary student confidence to appraise research has been associated with KT process efficiency [36]. This was a similar finding of this study and we believe this is the first survey of working agri-food public health veterinarians associating individual efficiency and stress with the process of keeping current. The relationship between KT barriers and stress in veterinarians is worth further investigation.

### Access

Swine veterinarian preference to consult with colleagues first for additional information is consistent with findings of other surveys [12, 17, 43, 53, 54], but swine veterinarians may differ in having greater preference to refer to journals. The distribution of relevant publications across journals complicates efforts to practice EBM for veterinarians with limited access to journal subscriptions. For example, over the last 30 years, IAV-S vaccine primary research has been published across 51 different journals, most of which are not open access or those preferred by respondents in this survey [51].

### Skill

Both topic and study design expertise is needed to assess validity [55–59]. Vaccine program management is a population medicine touchstone and understanding of the terms in this survey was assumed as fundamental [60–62]. Respondent overall low familiarity with R_0_ and Risk Ratio is worth further investigation, particularly if such short-comings translate into inappropriate extrapolations from vaccine efficacy or effectiveness studies to disparate field conditions. Understanding of types and causes of bias underpins skill to assess studies [63, 64]. Reported low familiarity with bias is consistent with finding of Vandeweerd et al. [12]. Lacking of familiarity with the term evidence pyramid was incongruent with high reported understanding of EBM. We did not explore however if this also equates with a low understanding of the hierarchical relationship of study design with susceptibility to bias.

### Critical appraisal versus information management

Consistent with previous surveys [36, 53, 65], the majority of respondents do not usually read methods sections of articles. By default then, it is reasonable to conclude that the majority also do not usually assess study validity, possibly also increasing the likelihood of assimilating biased information into practice. Although not a finding of this survey, student avoidance of methods sections was attributed to low confidence in critical appraisal skills [36].

Logically, recommendations for both human health and veterinary practitioners are to improve skills to critically appraise primary studies through training in the traditional skills of EBM; question formulation, comprehensive searching, determining relevance, critical assessment, synthesis, and presentation of findings [66–71]. While veterinarians report interest in such training [65, 72] emphasis on this approach might not be optimal for the profession. Training and maintenance of traditional EBM skills, through CE/CPD for example, while necessary and important, requires an investment of time, and considerable numerical literacy [16, 36, 73–76]. Opinions also differ on how to teach EBM/EBVM, and best methods for teaching are not clear [76]. Shurtz et al. [10] surveyed 22 U.S. and Canadian veterinary colleges and found no consistency in approaches to teaching EBVM skills within schools or between schools. Time, competing curriculum, perceived limited importance of EBVM, and the difficulty of teaching critical assessment, were the chief challenges to teaching EBVM skills [10, 36].

Alternatively, others recommend instead greater emphasis on teaching skills for information management over teaching traditional EBM skills [33, 34]. The rationale is that skill to quickly identify sources of relevant, synthesized, and contextualized information is highly advantageous in time constrained environments and more conducive to rapid end-user uptake of the best available research [5, 33, 34]. Importantly, however, the caveat is that robust and applicable syntheses are also readily accessible [5, 11, 34, 77].

### Syntheses versus primary research

It is noteworthy, considering reported challenges with time, skill, and access, that a significant proportion of respondents preferred individual primary research studies for staying current over expert summaries of the body of evidence. The reason for this preference is unknown. Low awareness of the advantages of using research syntheses, low awareness of available syntheses [10, 36, 78], few available syntheses, or difficulty accessing appropriate contextual or plain language summaries [79–81] may explain this finding. Alternatively, IPRPs may be sought based on authorship, to find research in support of clinical recommendations, or to review the methods reported in consideration of a future research study design; each of which may be problematic if there is collectively low professional awareness of issues with reliance on informal methods for selection of primary research [8, 82–87].

## Limitations

Participant self-selection and self-reporting may have biased results to reflect greater KT barriers. Experienced veterinarians may be over-represented; we did not explore association of years of practice with reported levels of skill, time or access. A 6% (80/1289) response rate is low and may not be representative of the source population. However demographics matched the AASV’s membership and conservative estimates suggest survey participants represented responses from veterinarians directly influencing a minimum of 2 million commercial pigs or 26% of the Canadian national sow herd [88], and approximately 9% of the US national sow herd [89]. Post survey, the epidemiological situation for African Swine Fever virus in Asia, and the diagnostic investigations related to Senecavirus A have changed possibly affecting updated rankings for these pathogens.

Disproportionate responses from Canadian versus overall member responses (~ 29% vs 6%) suggests advocacy may improve response; the CASV president encouraged participation at the CASV annual meeting, sending also three single purpose emailed reminders, as did Canadian regional swine association presidents both formally (group email) and informally. Lastly, the AASV Get-it For-Me service has since been discontinued.

## Conclusions

Swine veterinarians seek information predominantly on topics of endemic infectious diseases over other aspects of veterinary medicine, and colleagues, specialists and journals are important sources of information. The process of keeping up is stressful and not optimally efficient for many swine veterinarians. KT barriers are substantial and do not differ in large part from those for other veterinarians as reported in previous literature. This suggests a need to improve KT infrastructure. The current professional paradigm has veterinarians customizing their own path for keeping current using skills such as searching for, identifying, and appraising primary research. Findings also illustrate a need for additional training on epidemiologic principles, statistical methods, and interpretation of primary research, however notable potential shortcomings with emphasis on this strategy for CE/CPD were also discussed. Reasons why veterinarians choose to review primary research over syntheses is worth further evaluation. Consideration of this will be important in the planning of KT infrastructure improvements.

## Methods

### Survey timeline

The survey was launched online through the American Association of Swine Veterinarians (AASV) weekly e-letter to its membership on March 16, 2016, and closed April 26th, 2016.

### Survey development

The survey was generated using Qualtrics Survey Software, Copyright© 2016 Qualtrics, Provo, UT, USA. http://www.qualtrics.com available on license through the University of Guelph, Canada. Submissions were anonymous and no personal data were collected. Participation was incentivized with voluntary entry to a draw for a copy of Disease of Swine 10th edition (identities protected). The survey was offered in three languages (English, French and Spanish) and structured for online completion in 10 min or less via PC or mobile devices. The online survey tool was pre-tested in February 2016 by 10 experts geographically from Canada, the USA and the UK, in practitioner, academic, and government veterinary roles, and with expertise in swine veterinary medicine, and evidence based methodology.

### Question development

Full text for the survey tool is available in Additional file 2. Questions were grouped into three blocks; Block 1 identified interests, motivations, and priority information sources, Block 2 explored KT barriers of time, access, skill, and process, and format preferences for written information, Block 3 captured information on demographics and conference attendance. A differentiating question (Q23) in the Block 3 asked respondents to quantify their direct field oversight of swine production in terms of “sows worth of production”, which reflects influence over quantities of offspring entering the market. For all analyses of measures of association by veterinary role, respondents self-identifying as practitioners or reporting oversight of greater than 1000 sows worth of production were assigned the designated role of “direct oversight”, and respondents self-identifying as non-practitioners and reporting oversight of 1000 or fewer sows worth of production were assigned “indirect oversight” as their role.

All questions formatted as lists of options included also the option of ‘other’ and provision of open ended text boxes for elaboration. Likert scales (3, 4, and 5 point) were used for questions on opinions. Two questions addressed skill; self-report of familiarity with knowledge terms, and confidence level to appraise three aspects of research conduct (study design, choice of statistical analysis, and interpretation of findings). Knowledge terms were selected where understanding was believed pre-requisite to engagement in research appraisal. Terms were also selected as groupings with strong construct validity where understanding of one term implied that there would be understanding of the other terms in the group [64, 68]. Respondents were given a list of six options to explore preferred reading material length and format for keeping current with research; original primary research study, third party review of the individual study, or third party review of the body of evidence.

### Survey administration

The target population, the source population, and the sampling frame, respectively, were swine practitioners globally, the 2016 American Association of Swine Veterinarians (AASV) membership, and the AASV members subscribing to the AASV weekly e-letter. AASV members (1289) are involved in practice, industry and academia, in more than 40 countries [90]. Researchers sought prior approval and generated awareness for the survey with both the AASV and the Canadian Association of Swine Veterinarians (CASV) boards in early planning stages. A priori*,* no formal sample size calculation was performed understanding a 10% response rate was expected based on previous efforts to survey the membership, and that almost 100% of the membership received the e-Letter (AASV Associate Director, personal communication). Additional marketing included printed material displayed at the 47th AASV annual meeting registration table, and CASV president endorsement at the CASV annual board meeting. The survey link was posted in the e-Letter followed by three reminder notices at two week intervals, and remained accessible online for the duration of the survey period.

### Data entry and manipulation

Online submissions were collected using Qualtrics software and downloaded for analysis to Stata, StataCorp. 2015. Stata Statistical Software: Release 14. College Station, TX: StataCorp LP. Responses were excluded if respondents did not identify their role or self- identified as non-veterinarians.

### Statistical analysis

Proportions of responses were calculated both overall and stratified by designated veterinary role (as direct versus indirect). For the purposes of inferential analysis, categorical response options with more than two categories were aggregated into dichotomous outcomes (see Table 2 in Additional file 3). This was a post hoc decision due to the low survey response. Contingency tables were then used to estimate proportions by veterinary role and to generate prevalence ratios (PR) (and 95% confidence intervals). Fisher’s exact test was used to determine statistical significance of associations. Cronbach’s alpha was used to assess the internal consistency of responses to questions on self-reported level of skills to assess research [91, 92], with values ranging from 0.70 to 0.95 interpreted as acceptable [91].

## Supplementary information

**Additional file 1: Figure 1**. Timeline of Milestones for Veterinary Knowledge Translation Surveys, Evidence Based Medicine (EBM), and online (digital) information access. **Table 1**. Veterinary published surveys inclusive of a focus on KT barriers, veterinary information needs, or continuing education.

**Additional file 2.** Full copy of English version original Survey text of the Swine Veterinarian Infectious Disease Research Access Survey as launched March 2016 through the AASV e-Letter.

**Additional file 3: Table 1**. Demographics (%) of survey respondent by self-identified veterinary role. **Figure 1**. Demographic composition by role (%) of survey respondents versus the AASV membership. **Table 2A**. Summary of post hoc grouping of the multiple response options for each survey question into dichotomous responses for analysis (used in calculation of all prevalence ratios). **Table 2B**. Self-reported veterinary role as practitioner or non-practitioner versus dichotomized role. **Table 2C**. Self-reported veterinary role versus sows worth of veterinary oversight. **Table 2D**. Dichotomized veterinary role versus sows worth of veterinary oversight. **Table 3**. Dichotomised responses* for reported level of process efficiency (Q8) and level of process stress with staying current (Q9) versus role (direct vs. indirect). **Table 4**. (Q10) Self-reported familiarity with epidemiologic and evidence based terminology, by role. Response options not dichotomized (i.e the same information but dichotomized responses only are shown in Table 3 of main body of manuscript). **Table 5**. (Q12) Self -reported level of confidence to assess aspects of a research paper, by role (direct vs. indirect). **Table 6A**. (Q12) Self -reported level of confidence to assess aspects of a research paper, by role (direct vs. indirect) with responses dichotomized* as ‘Confident’ or ‘No Confidence/Do not evaluate’. **Table 6B.** (Q12) Association of having confidence* with usually reading* versus not usually reading the methods section of journal articles. **Table 7**. (Q4) Ranking of first (1st), second (2nd), and third (3rd) choices for getting more information for difficult clinical cases, by role (direct vs. indirect). **Table 8**. (Q11) Self-reported frequency of reading sections of scientific journal articles, by role. **Table 9**. Self-reported scientific journal access (Q15) and article service awareness (Q17) by frequency of blocked access (Q16) (Often, Occasionally, Rarely, Not at all). **Table 10**. (Q18) Association of selection of a preferred reading material format (for keeping current with a specific infectious disease topic) with veterinary role. **Table 11A.** (Q18) Respondent self-reported time*(Q7), skill*(Q10,12) and access*(Q11) by their choice of individual primary research papers (IPRP) as a preferred reading format for keeping current with a specific disease topic (IPRP not selected vs IPRP selected). **Table 11B**. (Q18) Association of time, skill and access for respondents with not selecting IPRP* versus selecting IPRP as a preferred reading format. **Table 12**. (Q18) Association of selected combinations of choices* of reading material format options with direct^†^ veterinary role versus indirect^††^ veterinary role.

## Data Availability

The datasets generated and/or analysed during the current study are not publicly available due to terms of the participant consent agreement, but are available from the corresponding author on reasonable request.

## Abbreviations

AASV: American Association of Swine Veterinarians
BOE: Body of evidence
CASV: Canadian Association of Swine Veterinarians
CE/CPD: Continuing education/continuing professional development
EAPHM: European Association for Porcine Health Management
EBM: Evidence Based Medicine
EBVM: Evidence Based Veterinary Medicine
IAV: Influenza A virus
IPRP: Individual primary research paper
JSHAP: Journal of Swine Health and Production
KT: Knowledge translation
PEDv: Porcine Epidemic Disease virus
PR: Prevalence ratio
PRRS: Porcine Reproductive and Respiratory Syndrome
