# Supplementary material for: Veterinarian barriers to knowledge translation (KT) within the context of swine infectious disease research: an international survey of swine veterinarians
Source: BMC Vet Res. 2020 Nov 2;16:416. doi: 10.1186/s12917-020-02617-8 (PMC7607664; doi:10.1186/s12917-020-02617-8)
Supplement: Supplementary file 1 — Additional file 1: Figure 1. Timeline of Milestones for Veterinary Knowledge Translation Surveys, Evidence Based Medicine (EBM), and online (digital) information access. Table 1. Veterinary published surveys inclusive of a focus on KT barriers, veterinary information needs, or continuing education. [file 12917_2020_2617_MOESM1_ESM.docx]

1^st^ swine multi-site isowean production system populated -1987

**SYREAF website ‘14**

**1972**

**Google incorporates ‘98**

Facebook incorporates ‘04, Twitter ‘07

**The Campbell Collaboration (C2) established ‘00**

**Archie Cochrane’s**

**Effectiveness and Efficiency ‘72**

Internet of Things (IoT) “invented” ‘99

**1970**

**2000**

**1990**

**2017**

“Web 1.0” World Wide Web proposed ‘89

“Big Data” term used in modern context mid 90’s

Commercial Internet born ‘74

**Penny & Penny 19**70

**Pelzer and Leysen 19**91

**Nielsen *et. al****.* 20 15

**Nielsen et. al.** 20 14

**Wales** 2000

**Haimerl *et al.*** 2013

**Vandeweerd *et. al****.* 2012

**Huntley *et. al.* 2017**

Penny 17

**Huntley *et. al.*** 2016

**Maes et al 2010**

Dale *et al.* 2013

**2017 Population 7.6 billion**

**1970 Population 3.7 billion**

**Cochrane Collaboration launched ‘92**

**Evidence Based Medicine defined ‘96**

**2010**

OIE established

<- 1924

“Stone Mountain Meeting” – 2010 **One Health** operationalized UN, World Bank recommend One Health

WHO guidance report on use of Mathematical Modelling in policy ‘16

“Web 2.0” term popularized for interactive/collaborative internet ‘04

**CEVM founded ‘09**

**Meehan & Harburg 2010**

Early Development of Open Access Journal Publishing

Moore et al 2000

Scopus and Google Scholar Bibliometric databases launched ‘04

**BestBETs for Vets website ‘13**

**Vet Record on EBVM ’98, Keene editorial on EBVM ‘00**

Dale *et al.* 2010

Dale *et al.* 2011

Delver 2008

Chun & Hwang ‘15

**EBVMA founded ‘04**

**SURVEYS**

**EBM**

**DIGITAL & DATA**

**Eldermire *et. al.* 2019**

Penny 17

**Marvin *et. al.* 2010**

Penny 17

**Figure 1. Timeline of Milestones for Veterinary Knowledge Translation Surveys, Evidence Based Medicine (EBM), and online (digital) information access.**

Relevance of veterinary surveys on sources of information prior to the rise of digital information in the early 2000’s may be limited. Evidence based veterinary medicine was first discussed by Malyicz, Fogle, and then Roper in the Veterinary Record during 1998 and again in an editorial by Bruce W. Keene in 2000 in J Vet Intern Med. 14:118-119. Research evidence methodologies were established with the Cochrane Collaboration in 1992, and the Campbell Collaboration in 2000.

**Table 1**. Veterinary published surveys inclusive of a focus on KT barriers, veterinary information needs, or continuing education

| **Authors** | **Year** | **Country** | **Survey focus*** | **Sample size**  (Response rate %) |
| --- | --- | --- | --- | --- |
| Penny & Penny[1] | 1978 | UK | Swine veterinarian interests, available research | 135/307 |
| Pelzer and Leysen[2] | 1991 | USA | Sources of information used by veterinarians (>80% exclusive small animal focus) | 272/548 |
| Wales[3] | 2000 | UK | Information seeking behaviour and use | n = 82 (39%) |
| Moore et al.[4] | 2000 | USA | Continuing education opinions and information sources | n=84 (focus group) |
| Delver[5] | 2008 | Canada | Continuing education needs and preferences | n=525 (54%) |
| Maes et al.[6] | 2010 | Belgium | Swine veterinarian practice general demographics (Abstract only in English – Full paper in Dutch) | n=43 |
| Marvin et al.[7] | 2010 | Canada | Ontario swine industry zoonotic knowledge and preferred information source | n=36 (veterinarians) |
| Meehan & Harburg[8] | 2010 | Australia | Small Animal Veterinary information sources | n=249 (22%) |
| Dale et al.[9] | 2010 | UK | Continuing education and veterinarian associations with learning preferences | n=775/2000 |
| Dale et al.[10] | 2011 | UK | Veterinarian /veterinary student survey of Web 2.0 use (focus group discussions) | n= 10+ 9 |
| Dale et al.[11] | 2013 | UK | Continued Professional Development motivation/barriers (from 2010 survey) | n=775/2000 |
| Vandeweerd et al.[12] | 2012 | Belgium | Private practitioners’ decision making process | n=201  (95%) |
| Haimerl et al.[13] | 2013 | Germany | Continuing education use and skills to evaluate evidence (Abstract only in English – Full paper in German) | n=293 |
| Nielsen et al.[14] | 2014 | UK | Veterinarian priority practice interests by role | n=4842/14,532 |
| Nielsen et al.[15] | 2015 | UK | Sources of information used by veterinarians | n=4842/14,532 |
| Chun and Hwang[16] | 2015 | South Korea | Continuing Education small animal program preferences | n=190/500 |
| Huntley et al.[17] | 2016 | International | Sources of information used by veterinarians | n=2137 |
| Huntley et al.[18] | 2017 | UK & International | Continued Professional Development Awareness of EBVM** | n=6310 |
| Eldermire et al.[19] | 2019 | North America | Veterinary student information seeking | n=226 |

*Unless otherwise stated, veterinarian population included all types of practice /species specializations

**EBVM = Evidence Based Veterinary Medicine.

Note: Literature was searched for additional relevant published veterinary surveys or questionnaires (Web of Science bibliographic platform and Google Scholar – Up to July 2016, abstract in English) using search strings with combinations and truncations of “veterinarian”, “survey”, “questionnaire”, “continuing education”, “knowledge translation”, and “information”. References from relevant papers were hand searched for missed studies.

1. Penny R, Penny J. Priorities for pig research: The results of the Second Pig Veterinary Society Questionnaire. In: Proceedings – The Pig Veterinary Society. 1978. p. 119–24.

2. Pelzer NL, Leysen JM. Use of information resources by veterinary practitioners. Bull Med Libr Assoc [Internet]. 1991;79(1):10–6. Available from: http://www.pubmedcentral.nih.gov/articlerender.fcgi?artid=225478&tool=pmcentrez&rendertype=abstract

3. Wales T. Practice makes perfect? Vets’ informatoin seeking behaviour and informatoin use explored. Aslib Proc. 2000;52(7):235–46.

4. Moore DA, Klingborg DJ, Brenner JS, Gotz AA. Perspectives in Professional Education in continuing veterinary medical education. J Am Vet Med Assoc. 2000;217(7):1001–6.

5. Delver HA. Continuing veterinary medical education needs and delivery preferences of Alberta veterinarians. J Vet Med Educ. 2008;35(1):129–37.

6. Maes D, Beken H vander, Dewulf J, Vliegher S de, Castryck F, Kruif A de. The functioning of the veterinarian in the Belgian pig sector: a questionnaire survey of pig practitioners. Vlaams Diergeneeskd Tijdschr. 2010;79(3):218–26.

7. Marvin DM, Dewey CE, Rajić A, Poljak Z, Young B. Knowledge of zoonoses among those affiliated with the ontario swine industry: A questionnaire administered to selected producers, allied personnel, and veterinarians. Foodborne Pathog Dis. 2010;7(2):159–66.

8. Meehan MP, Harburg KN. Information sources used by Veterinarians and their perceptions of the ASAVA and the AVP. Aust Vet Pract. 2010;40(3):109–18.

9. Dale VHM, Pierce SE, May SA. The Importance of Cultivating a Preference for Complexity in Veterinarians for Effective Lifelong Learning. J Vet Med Educ [Internet]. 2010;37(2):165–71. Available from: http://jvme.utpjournals.press/doi/10.3138/jvme.37.2.165

10. Dale VHM, Kinnison T, Short N, May SA, Baillie S. Web 2.0 and the veterinary profession: Current trends and future implications for Lifelong Learning. Vet Rec. 2011;169(18).

11. Dale VHM, Pierce SE, May S a. Motivating factors and perceived barriers to participating in continuing professional development: a national survey of veterinary surgeons. Vet Rec [Internet]. 2013;173:247. Available from: http://www.ncbi.nlm.nih.gov/pubmed/23980235

12. Vandeweerd J-M, Vandeweerd S, Gustin C, Keesemaecker G, Cambier C, Clegg P, et al. Understanding Veterinary Practitioners’ Decision-Making Process: Implications for Veterinary Medical Education. J Vet Med Educ [Internet]. 2012;39(2):142–51. Available from: http://jvme.utpjournals.press/doi/10.3138/jvme.0911.098R1

13. Haimerl P, Arlt S, Heuwieser W. Entscheidungsfindung in der tierartlichen Praxis. Tierarztl Prax Kleintiere. 2013;4:229–36.

14. Nielsen TD, Dean RS, Robinson NJ, Massey A, Brennan ML. Survey of the UK veterinary profession: Common species and conditions nominated by veterinarians in practice. Vet Rec. 2014;174(13):324.

15. Nielsen TD, Dean RS, Massey A, Brennan ML. Survey of the UK veterinary profession 2: Sources of information used by veterinarians. Vet Rec. 2015;177(7).

16. Chun M-S, Hwang C-Y. Continuing Veterinary Medical Education Needs Assessment of Small Animal Practitioners in South Korea. J Vet Med Educ. 2015;42(3):232–8.

17. Huntley SJ, Dean RS, Massey A, Brennan ML. International evidence-based medicine survey of the veterinary profession: Information sources used by veterinarians. PLoS One [Internet]. 2016;11(7):1–18. Available from: http://dx.doi.org/10.1371/journal.pone.0159732

18. Huntley S, Dean R, Brennan M. The Awareness of the International Veterinary Profession of Evidence-Based Veterinary Medicine and Preferred Methods of Training. Vet Sci [Internet]. 2017;4(1):15. Available from: http://www.mdpi.com/2306-7381/4/1/15

19. Eldermire ERB, Fricke S, Alpi KM, Davies E, Kepsel AC, Norton HF. Information seeking and evaluation: A multi-institutional survey of veterinary students. J Med Libr Assoc. 2019;107(4):515–26.
