# Supplementary material for: Veterinarian barriers to knowledge translation (KT) within the context of swine infectious disease research: an international survey of swine veterinarians
Source: BMC Vet Res. 2020 Nov 2;16:416. doi: 10.1186/s12917-020-02617-8 (PMC7607664; doi:10.1186/s12917-020-02617-8)
Supplement: Supplementary file 2 — Additional file 2. Full copy of English version original Survey text of the Swine Veterinarian Infectious Disease Research Access Survey as launched March 2016 through the AASV e-Letter. [file 12917_2020_2617_MOESM2_ESM.docx]

**Additional File 2.** Full copy of English version original Survey text of the Swine Veterinarian Infectious Disease Research Access Survey as launched March 2016 through the AASV e-Letter. (DOCX)

Consent Page:

Welcome and thank you for your interest. This survey is available also in French and Spanish. You may choose your preferred language by clicking the language selection button in the top right corner. Survey results will help our work developing methods for improved access to veterinary research.  Please know all submissions are anonymous.  No names, IP addresses, or personal identifiers will be collected or used in reports, presentations or publications. Results will be presented in aggregate only.   We strive for complete confidentially but because of the online format it can't be guaranteed. You are free to skip any questions and you can exit the survey at any time by closing the survey window.  Due to responses being anonymous and therefore unidentifiable, they cannot be withdrawn once submitted.  Findings will be posted later this year in the AASV e-Letter. If you have any questions please feel free to contact Sheila Keay, DVM, MBA, MPH, PhD student (Faculty Advisor - Zvonimir Poljak, DVM, MSc, PhD), Ontario Veterinary College, University of Guelph, Guelph, Canada [*current contact information provided here*]. This survey (REB# 16JA029) is funded from a research fellowship made available through the Ontario Veterinary College and it has been reviewed by the Research Ethics Board for compliance with federal guidelines for research involving human participants.  If you have questions or concerns regarding your rights and welfare as a research participant in this study please contact: Director, Research Ethics; University of Guelph; reb@uoguelph.ca; (519) 824-4120 (ext. 56606). At the end of the survey you will be asked if you would like to participate in a draw for a 10th Edition of Diseases of Swine, valued at $250.   Participation in the draw will not be anonymous but will be separate and not linked to your survey submission to maintain anonymity of survey submissions. A copy of this consent and contact form may be printed by clicking on the print button below.   By selecting “I agree to participate” below you confirm that you understand:   Participation is voluntary, anonymous, and you can withdraw at any time. You do not waive any of your AASV / CASV membership rights and there are no foreseeable risks for participating in this survey.

- I agree to participate.

In the following 6 questions, please tell us about your swine health interests.

Q1 What general topics do you most often seek swine information for? Please select up to three (3) choices.

- Antibiotics and other therapeutics
- Antimicrobial resistance
- Biosecurity (bioexclusion)
- Disease control / elimination strategies (biocontainment/ biomanagement)
- Disinfectants
- Emerging or new swine disease information
- Environment (heating, ventilation, etc.)
- Genetics
- Nutrition
- Reproduction
- Vaccines
- Welfare
- Zoonoses
- Other (If other please specify in the space below) ____________________

Q2 Which swine viruses do you most often seek information for? Please select up to three (3).

- African swine fever virus
- Classical swine fever virus
- Foot-and-mouth disease virus
- Influenza A virus of swine
- Porcine circovirus type 2
- Porcine epidemic diarrhea virus
- Porcine reproductive and respiratory syndrome virus
- Porcine rotavirus
- Pseudorabies virus
- Senecavirus A
- Other (If other viruses please specify in the space below) ____________________

Q3 Which swine bacteria do you most often seek information for? Please select up to three (3).

- Actinobacillus pleuropneumoniae
- Brachyspira hyodysenteriae
- Escherichia coli
- Haemophillus parasuis
- Lawsonia intracellularis
- Mycoplasma hyopneumoniae
- Salmonella
- Streptococcus suis
- Other (If other bacteria or other pathogens please specify in the space below) ____________________

Q4 When you have a difficult clinical case what is your first, second, and third choice for getting more information?

|  | Ask a colleague or another vet (1) | Contact a specialist (2) | Scientific journals (6) | Conference attendance (3) | Conference proceedings (5) | General internet search (9) | Industry magazines (7) | Textbooks (8) | Manuals written by veterinary organizations (13) | AASV-L-Digest (10) | Veterinary websites or blogs (11) | Other (12) |
| --- | --- | --- | --- | --- | --- | --- | --- | --- | --- | --- | --- | --- |
| First choice (1) |  |  |  |  |  |  |  |  |  |  |  |  |
| Second choice (14) |  |  |  |  |  |  |  |  |  |  |  |  |
| Third choice (3) |  |  |  |  |  |  |  |  |  |  |  |  |

Q5 In question 4 above  if "other" was selected as your first, second and/or third choice for source of information please specify in the space below, otherwise skip to question 6.

Q6 What motivates you the most to seek infectious disease information?

- Difficult case management when diagnosis is confirmed
- Difficult case management when diagnosis is not confirmed
- Awareness of emerging diseases
- Other work requirement (e.g. research or report writing)
- Required continuing education for license
- General interest
- Other (if other please specify in the space below) ____________________

The next 12 questions focus on swine infectious disease research information and how you use it.

Q7 On average, approximately how much time do you spend each week on staying current or for reviewing infectious disease research information?

- 0
- >0 - 30 minutes
- >30 minutes - 1 hour
- > 1 hour - 3 hours
- > 3 hours

Q8 Do you have a good process for staying current with swine infectious disease research? Please indicate the efficiency of your process.

|  | Complete efficiency (1) (1) | 2 (2) | 3 (3) | 4 (5) | No efficiency (5) (6) |
| --- | --- | --- | --- | --- | --- |
| Level of efficiency of your process (1) |  |  |  |  |  |

Q9 Is it stressful for you to stay current on swine infectious disease research? Please indicate your level of stress with staying current.

|  | No Stress (1) (1) | 2 (4) | 3 (2) | 4 (7) | Completely stressful (5) (8) |
| --- | --- | --- | --- | --- | --- |
| Level of Stress (1) |  |  |  |  |  |

Q10 How familiar are you with the following terms?

|  | Can explain (1) | Have heard of (2) | Not familiar (3) |
| --- | --- | --- | --- |
| Evidence based medicine (EBM) (1) |  |  |  |
| Evidence-based veterinary medicine (EBVM) (2) |  |  |  |
| Systematic review (3) |  |  |  |
| Meta-analysis (4) |  |  |  |
| Evidence pyramid (5) |  |  |  |
| Selection bias (6) |  |  |  |
| Information bias (7) |  |  |  |
| Confounding bias (14) |  |  |  |
| Risk ratio (9) |  |  |  |
| Vaccine efficacy (10) |  |  |  |
| Vaccine effectiveness (11) |  |  |  |
| Basic reproduction number [R0] (12) |  |  |  |

Q11 Please indicate how you read the parts of a scientific paper you have selected to review.

|  | Usually (1) | Occasionally (2) | Rarely or not at all (3) |
| --- | --- | --- | --- |
| Abstract (1) |  |  |  |
| Introduction (2) |  |  |  |
| Materials and Methods (3) |  |  |  |
| Results (4) |  |  |  |
| Discussion (5) |  |  |  |
| Conclusion/Implications (6) |  |  |  |
| Conflict of Interest (7) |  |  |  |
| Acknowledgements (8) |  |  |  |

Q12 Please indicate your level of confidence to evaluate in a research paper the appropriateness of the following:

|  | Complete confidence (1) | Some confidence (2) | No confidence (3) | I do not evaluate this (6) |
| --- | --- | --- | --- | --- |
| Study design used (1) |  |  |  |  |
| Statistical methods used (2) |  |  |  |  |
| Statistical interpretation of results (3) |  |  |  |  |

Q13 What do you use most frequently to conduct a search for swine infectious disease research information? Please select up to two (2).

- Google or Google Scholar (1)
- Bibliographic databases such as MEDLINE, Agricola or PubMed (2)
- AASV Swine Information Library (4)
- References and/or bibliography from published studies (12)
- I ask colleagues to forward recommended references (13)
- Other (if other, please specify in the space below) (5) ____________________

Q14 What scientific journals (excluding conference proceedings) do you use most often to get swine infectious disease research information? Please select up to three (3).

- Canadian Veterinary Journal
- Journal of Animal Science
- Journal of Swine Health and Production
- Journal of the American Veterinary Medical Association
- Journal of Veterinary Diagnostic Investigation
- Livestock Science
- Preventive Veterinary Medicine
- Porcine Health Management
- The Veterinary Record
- Veterinary Clinics of North America
- Veterinary Research
- Other (if other, please list their title(s) in the space below) ____________________

Q15 How many scientific journals do you have subscription access to (either personal subscriptions or subscriptions held at your place of work)?

- 0
- 1-2
- 3-4
- 5-6
- >6

Q16 How often are you unable to access full research papers due to lacking a subscription (i.e. only have access to the abstract and not the full text) ?

- Often
- Occasionally
- Rarely
- Not at all

Q17 Are you aware of the AASV Get it For Me Service?

- Yes
- No

Q18 What would you most prefer to read to help you to keep current with research on specific disease topics? Please select up to two (2).

- Individual published primary research papers
- A one (1) page expert critical summary of the individual primary research paper
- A full narrative expert critical review report on the body of evidence
- A three (3) page summary of an expert critical review of the body of evidence
- A one (1) page summary of an expert critical review of the body of evidence
- Other (if other please specify in the space below) ____________________

Almost done. These final 6 questions help us understand the survey demographic.

Q19 In which country or region do you primarily work?

- U.S.A.
- Canada
- Mexico
- Europe
- Asia
- Central/ South America
- Australia/ New Zealand
- Africa

Q20 What is your involvement in the pork industry?

- Practitioner (work in private practice or within a commercial swine production system)
- Allied Corporate Veterinarian (work within the allied pork industry (i.e. feed, pharmaceutical, breeding stock, etc.)
- Public Veterinarian defined as working within government or public health care systems
- Research or extension Veterinarian (work at a university or teaching institution)
- Veterinary undergraduate student
- Veterinary graduate student
- Retired veterinarian
- Non-veterinarian researcher
- Non-veterinarian other

Q21 How many years have you been working with swine?

- <1--4 years
- >4-10 years
- >10-15 years
- > 15 years

Q22 Approximately what percentage of your work is focused on swine?

- 0
- >0-25%
- >25%-50%
- >50%-75%
- >75%-99%
- 100%

Q23 Approximately how many sows worth of production do you provide with direct veterinary services?

- 0
- >0-1000
- >1000-5,000
- >5,000-50,000
- >50,000-100,000
- >100,000

Q24 Have you attended a veterinary conference in the last two years where swine infectious disease research information was presented?

- Yes
- No
