# Supplementary material for: Veterinarian barriers to knowledge translation (KT) within the context of swine infectious disease research: an international survey of swine veterinarians
Source: BMC Vet Res. 2020 Nov 2;16:416. doi: 10.1186/s12917-020-02617-8 (PMC7607664; doi:10.1186/s12917-020-02617-8)
Supplement: Supplementary file 3 — Additional file 3: Table 1. Demographics (%) of survey respondent by self-identified veterinary role. Figure 1. Demographic composition by role (%) of survey respondents versus the AASV membership. Table 2A. Summary of post hoc grouping of the multiple response options for each survey question into dichotomous responses for analysis (used in calculation of all prevalence ratios). Table 2B. Self-reported veterinary role as practitioner or non-practitioner versus dichotomized role. Table 2C. Self-reported veterinary role versus sows worth of veterinary oversight. Table 2D. Dichotomized veterinary role versus sows worth of veterinary oversight. Table 3. Dichotomised responses* for reported level of process efficiency (Q8) and level of process stress with staying current (Q9) versus role (direct vs. indirect). Table 4. (Q10) Self-reported familiarity with epidemiologic and evidence based terminology, by role. Response options not dichotomized (i.e the same information but dichotomized responses only are shown in Table 3 of main body of manuscript). Table 5. (Q12) Self -reported level of confidence to assess aspects of a research paper, by role (direct vs. indirect). Table 6A. (Q12) Self -reported level of confidence to assess aspects of a research paper, by role (direct vs. indirect) with responses dichotomized* as ‘Confident’ or ‘No Confidence/Do not evaluate’. Table 6B. (Q12) Association of having confidence* with usually reading* versus not usually reading the methods section of journal articles. Table 7. (Q4) Ranking of first (1st), second (2nd), and third (3rd) choices for getting more information for difficult clinical cases, by role (direct vs. indirect). Table 8. (Q11) Self-reported frequency of reading sections of scientific journal articles, by role. Table 9. Self-reported scientific journal access (Q15) and article service awareness (Q17) by frequency of blocked access (Q16) (Often, Occasionally, Rarely, Not at all). Table 10. (Q18) Association of selection o [file 12917_2020_2617_MOESM3_ESM.docx]

**Additional File 3** (DOCX)

**Table 1.**  Demographics (%) of survey respondent by self-identified veterinary role.

**Figure 1.** Demographic composition by role (%) of survey respondents versus the AASV membership.

**Table 2A.** Summary of *post hoc* grouping of the multiple response options for each survey question into dichotomous responses for analysis (used in calculation of all prevalence ratios).

**Table 2B.** Self-reported veterinary role as practitioner or non-practitioner versus dichotomized role

**Table 2C.** Self-reported veterinary role versus sows worth of veterinary oversight

**Table 2D**. Dichotomized veterinary role versus sows worth of veterinary oversight

**Table 3**. Dichotomised responses* for reported level of process efficiency (Q8) and level of process stress with staying current (Q9) versus role (direct vs. indirect).

**Table 4**. (Q10) Self-reported familiarity with epidemiologic and evidence based terminology, by role. Response options not dichotomized (i.e the same information but dichotomized responses only are shown in Table 3 of main body of manuscript)

**Table 5. (Q12)** Self -reported level of confidence to assess aspects of a research paper, by role (direct vs. indirect).

**Table 6A**. (Q12) Self -reported level of confidence to assess aspects of a research paper, by role (direct vs. indirect) with responses dichotomized* as ‘Confident’ or ‘No Confidence/Do not evaluate’.

**Table 6B. (Q12)** Association of having confidence* with usually reading* versus not usually reading the methods section of journal articles

**Table 7**. **(Q4)** Ranking of first (1st), second (2nd), and third (3rd) choices for getting more information for difficult clinical cases, by role (direct vs. indirect).

**Table 8**. **(Q11)** Self-reported frequency of reading sections of scientific journal articles, by role. **Table 9**. Self-reported scientific journal access **(Q15)** and article service awareness **(Q17)** by frequency of blocked access **(Q16)** (Often, Occasionally, Rarely, Not at all)

**Table 10**. (Q18) Association of selection of a preferred reading material format (for keeping current with a specific infectious disease topic) with veterinary role.

**Table 11A.** (Q18) Respondent self-reported time*(Q7), skill*(Q10,12) and access*(Q11) by their choice of individual primary research papers (IPRP) as a preferred reading format for keeping current with a specific disease topic (IPRP not selected vs IPRP selected)

**Table 11B**. **(Q18)** Association of time, skill and access for respondents with not selecting IPRP* versus selecting IPRP as a preferred reading format

**Table 12**. **(Q18)** Association of selected combinations of choices* of reading material format options with direct^†^ veterinary role versus indirect^††^ veterinary role

**Table 1**. Demographics (%) of survey respondent by self-identified veterinary role.

|  | **Practitioner*** | **Allied**† | **Research/ Ext.** Ŧ | **Public** †† | **Other**††† | **Overall** |
| --- | --- | --- | --- | --- | --- | --- |
|  | % | % | % | % | % | % |
| **Primary country (region) of work** | |  |  |  |  |  |
| U.S.A. (n=31) | 38 | 35 | 55 | 27 | 50 | 39 |
| Canada (n=40) | 49 | 47 | 45 | 73 | 25 | 50 |
| Other** (n=9) | 14 | 18 | 0 | 0 | 25 | 11 |
| **Total (n=80)** | 100 | 100 | 100 | 100 | 100 | 100 |
| **Years working with swine** | |  |  |  |  |  |
| <1-4yrs (n=11) | 19 | 6 | 27 | 0 | 0 | 14 |
| >4-10yrs (n=7) | 8 | 6 | 0 | 27 | 0 | 9 |
| >10-15yrs (n=11) | 14 | 18 | 18 | 9 | 0 | 14 |
| >15yrs (n=51) | 59 | 71 | 55 | 64 | 100 | 64 |
| **Total (n=80)** | 100 | 100 | 100 | 100 | 100 | 100 |
| **Percentage of work focused on swine** | | |  |  |  |  |
| >0-25% (n=14) | 11 | 6 | 18 | 55 | 25 | 18 |
| >25%-50% (n=4) | 0 | 0 | 9 | 27 | 0 | 5 |
| >50%-75% (n=6) | 3 | 12 | 9 | 9 | 25 | 8 |
| >75%-99% (n=15) | 22 | 18 | 27 | 9 | 0 | 19 |
| 100% (n=41) | 65 | 65 | 36 | 0 | 50 | 51 |
| **Total (n=80)** | 100 | 100 | 100 | 100 | 100 | 100 |
| **Approximate sows worth of production providing with direct veterinary service (000s)** | | | | | | |
| 0 (n=31) | 8 | 53 | 64 | 91 | 67 | 39 |
| >0-1000 (n=5) | 3 | 0 | 27 | 0 | 33 | 6 |
| >1000-5,000 (n=6) | 11 | 6 | 0 | 9 | 0 | 8 |
| >5,000-50,000 (n=22) | 54 | 6 | 9 | 0 | 0 | 28 |
| >50,000-100,000 (n=6) | 16 | 0 | 0 | 0 | 0 | 8 |
| >100,000 (n=9) | 8 | 35 | 0 | 0 | 0 | 11 |
| **Total (n=79)** | 100 | 100 | 100 | 100 | 100 | 100 |

*Practitioner: work in private practice or within a commercial swine production system, †Allied Corporate Veterinarian: work within the allied pork industry (i.e. feed, pharmaceutical, breeding stock, etc.), Ŧ Research or Extension Veterinarian: work at a university or teaching institution, †† Public Veterinarian: working within government or public health care systems, ††† Other Veterinarian: Retired(3), graduate student(1), **Other countries/regions: Mexico (1) Europe (4) Asia (1) Central/South America (1) Australia/New Zealand (3)

**Figure 1.** Demographic composition by role (%) of survey respondents versus the AASV membership.

Source: AASV 2017 Membership Directory

Practitioner: work in private practice or within a commercial swine production system, Allied Veterinarian: work within the allied pork industry (e.g. feed, pharmaceutical, or breeding stock companies), Research or Extension Veterinarian: work at a university or teaching institution, Public Veterinarian: working within government or public health care systems, Other Veterinarian: Retired, graduate student

**Table 2A.** Summary of *post hoc* grouping of the multiple response options for each survey question into dichotomous responses for analysis (used in calculation of all prevalence ratios).

| Q | **Categorical response options as available in the survey question** | **Options collapsed into dichotomous responses (1,0) for analysis** |
| --- | --- | --- |
| 4 | First choice Go to for information – multiple options Colleague, specialist, journals, conference, internet, magazines, manuals, AASV L-digest, veterinary websites, other | 1^st^ choice colleague (1) or other than colleague (0) |
|  |  | 1^st^ choice scientific journals (1) or other than scientific journals (0) |
| 6 | Difficult case with diagnosis, difficult case without diagnosis, emerging disease, other work, continuing education, general interest, other | ‘*Top motivator difficult case’* (0) or ‘*other than difficult case*’(1) |
| 7 | 0, >0-30 min, >30 min – 1hr, >1hr-3 hrs, >3hrs | 0-1 hour (1), greater than 1hour (0) |
| 8 | Likert scale 1 to 5 with 1=*Complete efficiency* and 5= *No efficiency* (combined 1&2= High; 3,4&5=Low) | ‘*High efficiency’* (0) – grouping responses of 1 and 2  ‘*moderate to no efficiency*’ (1) - grouping response of 3, 4 and 5 |
| 8 | Likert scale 1 to 5 with 1=*Complete efficiency* and 5= *No efficiency* (combined 1,2&3= Moderate to High;4&5=Low) | ‘*Moderate to high efficiency*’(0) – grouping responses of 1,2 and 3  ‘*low to no efficiency’*(1) |
| 9 | Likert scale 1 to 5 with 1=*No stress* and 5=*Completely stressful* (combined 1&2=Low; 3,4&5=High) | *‘Low to no stress’* (0) – grouping responses of 1 and 2  ‘*moderate to completely stressful’* (1) - grouping response of 3, 4 and 5 |
| 9 | Likert scale 1 to 5 with 1=*No stress* and 5=*Completely stressful* (combined 1,2&3=Moderate to Low; 4&5=High) | ‘*Moderate to low stress*’ (0) – grouping responses of 1,2 and 3  ‘*high to completely stressful’* (1) – grouping responses of 4 and 5 |
| 10 | *Can explain, have heard of*, *not familiar* (combined *have heard of* and *not familiar*, into ‘*cannot explain*’) | ‘*Can explain’*(1) or ‘*cannot explain*’ (0) confounding bias |
|  |  | ‘*Can explain’*(1) or ‘*cannot explain*’ (0)selection bias |
|  |  | ‘*Can explain’*(1) or ‘*cannot explain*’ (0) information bias |
|  |  | ‘*Can explain’*(1) or ‘*cannot explain*’ (0) evidence pyramid |
|  |  | ‘*Can explain’*(1) or ‘*cannot explain*’ (0) basic reproductive number |
| 11 | *Usually, Occasionally, Rarely or not at all*  (combined *Occasionally* and *Rarely or not at all*, into ‘*not usually’*) | ‘*Usually read*’(1) or ‘*do not usually read’*(0) methods and materials |
|  |  | ‘*Usually read*’(1) or ‘*do not usually read’* (0) results section |
| 12 | *Complete confidence, some confidence, no confidence, do not evaluate* (combined: *Complete confidence* + *some confidence* = ‘confident’  *No confidence* + *do not evaluate* = ‘*No confidence /do not evaluate’*) | ‘*No confidence /do not evaluate*’ (1), or ‘*Confident’* (0) to evaluate appropriateness of statistical methods used |
|  |  | ‘*No confidence /do not evaluate*’ (1), or ‘*Confident’* (0) to evaluate appropriateness of statistical interpretation of results |
|  |  | ‘*No confidence /do not evaluate*’ (1), or ‘*Confident’* (0) to evaluate appropriateness of study design used |
| 15 | Number of journals with subscription access 0, 1-2, 3-4, 5-6, >6 | Access to 0-6 journals (0), >6 (1) |
| 16 | Frequency of blocked article access as *Often, Occasionally, Rarely,* or *Not at all* (combined *Occasionally, rarely*, and *Not at all* = ‘*not often’*) | Access blocked *Often* (1), Access blocked ‘*not often’*(0) |
| **20**  **&**  **23** | **Role**: Practitioner, Allied Corporate, Public, Research or Extension, Student (graduate and undergraduate), Retired, Non-veterinary researcher, Non-veterinary other  **Q23 (Sows worth of production; 0, >0-1K, >1K-5K, >5K-50K, >50K-100K, >100K)** | *‘Direct’* (1), ‘*Indirect’* (0) **Conditioned on**: Q20 (self-identified as a practitioner or a non-practitioner) and on Q23 (sows worth of production) (See Tables 2B, 2C, and 2D)  Direct = self-identified practitioner OR a non-practitioner that provides >1000 sows worth of direct veterinary services (See Table 2C); Indirect = self-identified non-practitioner that provides 0 to 1000 sows worth with direct veterinary services.(See Table 2D) |
|  |  |  |

**Table 2B.** Self-reported veterinary role as practitioner or non-practitioner versus dichotomized role

| Self-reported veterinary role (Q20) | **Veterinary Role** (dichotomized₮) | | |
| --- | --- | --- | --- |
|  | **Direct** | **Indirect** | Total |
| Practitioner | 37 | 0 | 37 |
| Non-practitioner | **10** | 32 | 42 |
| Total | **47** | **32** | **79** |

**Table 2C.** Self-reported veterinary role versus sows worth of veterinary oversight

| Self-reported veterinary role (Q20) | Reported # of sows worth of production‡ respondent provides with direct veterinary services (Q23) | | | | | |  |
| --- | --- | --- | --- | --- | --- | --- | --- |
|  | 0 | >0-1000 | >1000-5000 | >5000-50,000 | >50,000-100,000 | >100,000 | Total |
| Practitioner | 3 | 1 | 4 | 20 | 6 | 3 | 37 |
| Non-practitioner | 28 | 4 | **2** | **2** | **0** | **6** | 42 |
| Total | 31 | 5 | 6 | 22 | 6 | 9 | 79 |

**Table 2D.** Dichotomized veterinary role versus sows worth of veterinary oversight

| **Veterinary role** (dichotomised₮) | Reported #of sows worth of production‡ respondent provides with direct veterinary services (Q23) | | | | | |  |
| --- | --- | --- | --- | --- | --- | --- | --- |
|  | 0 | >0-1000 | >1000-5000 | >5000-50,000 | >50,000-100,000 | >100,000 | Total |
| **Direct** | 3 | 1 | 6 | 22 | 6 | 9 | **47** |
| **Indirect** | 28 | 4 | 0 | 0 | 0 | 0 | **32** |
| Total | 31 | 5 | 6 | 22 | 6 | 9 | **79** |

₮ based on self-reported practitioner role and on reported number of sows worth of veterinary oversight where respondents not identifying as practitioners also reported providing direct veterinary health oversight for a substantial number of sows worth of production (see blue circle in Table 2C). This demographic was therefore also aggregated into the grouping of a ‘direct’ veterinary role in Table 2B.

‡ Note: 1000 sows worth of production equals approximately 20,000 market offspring/year

**Table 3**. Dichotomised responses* for reported level of process efficiency (Q8) and level of process stress with staying current (Q9) versus role (direct vs. indirect).

|  | Indirect Oversight† | | Direct Oversight†† | | Overall | |
| --- | --- | --- | --- | --- | --- | --- |
|  | % | 95% CI | % | 95% CI | % | 95% CI |
| Q8. Process efficiency to stay current (high) |  |  |  |  |  |  |
| High to complete efficiency (1,2) (n=30) | 38 | [22,55] | 38 | [25,53] | 38 | [28,49] |
| Moderate to low efficiency (3,4,5) (n=49) | 63 | [45,78] | 62 | [47,75] | 62 | [51,72] |
| Q9. Process stress to stay current (moderate/high) |  |  |  |  |  |  |
| Low stress (1,2) (n=32) | 38 | [22,55] | 43 | [29,57] | 41 | [30,52] |
| Moderate to high stress (3,4,5)(n=47) | 63 | [45,78] | 57 | [43,71] | 59 | [48,70] |
| Total (n=79) | 100 |  | 100 |  | 100 |  |

† Indirect oversight = non-practitioner that provides 1000 sows worth or less with direct veterinary services.

†† Direct oversight = practitioner or a non-practitioner that provides >1000 sows worth with direct veterinary services.

* Multiple response options for questions grouped into dichotomous options (See Table 2A in Additional File 3)

**Table 4**. **(Q10)** Self-reported familiarity with epidemiologic and evidence based terminology, by role. Response options not dichotomized (i.e the same information but dichotomized responses only are shown in Table 3 of main body of manuscript)

|  | **Indirect Oversight†** | | **Direct Oversight††** | | **Overall** | |
| --- | --- | --- | --- | --- | --- | --- |
|  | Column % | 95% CI | Column % | 95% CI | Column % | 95% CI |
| **Evidence based medicine (EBM)** | |  |  |  |  |  |
| Can explain (n=56) | 72 | [54,85] | 70 | [55,82] | 71 | [60,80] |
| Have heard of (n=23) | 28 | [15,46] | 30 | [18,45] | 29 | [20,40] |
| **Evidence-based veterinary medicine (EBVM)** | | |  |  |  |  |
| Can explain (n=57) | 69 | [51,83] | 74 | [60,85] | 72 | [61,81] |
| Have heard of (n=20) | 25 | [13,43] | 26 | [15,40] | 25 | [17,36] |
| Not familiar (n=2) | 6 | [2,22] | 0 |  | 3 | [1,10] |
| **Systematic review** |  |  |  |  |  |  |
| Can explain (n=47) | 59 | [42,75] | 60 | [45,73] | 59 | [48,70] |
| Have heard of (n=29) | 38 | [22,55] | 36 | [24,51] | 37 | [27,48] |
| Not familiar (n=3) | 3 | [0,20] | 4 | [1,16] | 4 | [1,11] |
| **Meta-analysis** |  |  |  |  |  |  |
| Can explain (n=46) | 66 | [48,80] | 53 | [39,67] | 58 | [47,69] |
| Have heard of (n=27) | 25 | [13,43] | 40 | [27,55] | 34 | [24,46] |
| Not familiar (n=6) | 9 | [3,26] | 6 | [2,18] | 8 | [3,16] |
| **Evidence pyramid** |  |  |  |  |  |  |
| Can explain (n=16) | 19 | [9,36] | 22 | [12,36] | 21 | [13,31] |
| Have heard of (n=34) | 28 | [15,46] | 54 | [40,68] | 44 | [33,55] |
| Not familiar (n=28) | 53 | [36,70] | 24 | [14,39] | 36 | [26,47] |
| **Selection bias** |  |  |  |  |  |  |
| Can explain (n=47) | 66 | [48,80] | 55 | [41,69] | 59 | [48,70] |
| Have heard of (n=21) | 19 | [9,36] | 32 | [20,47] | 27 | [18,38] |
| Not familiar (n=11) | 16 | [7,33] | 13 | [6,26] | 14 | [8,24] |
| **Information bias** |  |  |  |  |  |  |
| Can explain (n=39) | 56 | [39,72] | 45 | [31,59] | 49 | [38,60] |
| Have heard of (n=31) | 31 | [17,49] | 45 | [31,59] | 39 | [29,51] |
| Not familiar (n=9) | 13 | [5,29] | 11 | [4,23] | 11 | [6,21] |
| **Confounding bias** |  |  |  |  |  |  |
| Can explain (n=38) | 53 | [36,70] | 45 | [31,59] | 48 | [37,59] |
| Have heard of (n=27) | 25 | [13,43] | 40 | [27,55] | 34 | [24,46] |
| Not familiar (n=14) | 22 | [11,40] | 15 | [7,28] | 18 | [11,28] |
| **Risk ratio** |  |  |  |  |  |  |
| Can explain (n=45) | 63 | [45,78] | 53 | [39,67] | 57 | [46,68] |
| Have heard of (n=25) | 25 | [13,43] | 36 | [24,51] | 32 | [22,43] |
| Not familiar (n=9) | 13 | [5,29] | 11 | [4,23] | 11 | [6,21] |
| **Vaccine efficacy** |  |  |  |  |  |  |
| Can explain (n=69) | 84 | [67,93] | 89 | [77,96] | 87 | [78,93] |
| Have heard of (n=9) | 13 | [5,29] | 11 | [4,23] | 11 | [6,21] |
| Not familiar (n=1) | 3 | [0,20] | 0 |  | 1 | [0,9] |
| **Vaccine effectiveness** |  |  |  |  |  |  |
| Can explain (n=60) | 72 | [54,85] | 79 | [64,88] | 76 | [65,84] |
| Have heard of (n=15) | 19 | [9,36] | 19 | [10,33] | 19 | [12,29] |
| Not familiar (n=4) | 9 | [3,26] | 2 | [0,14] | 5 | [2,13] |
| **Basic reproduction number [R0]** | |  |  |  |  |  |
| Can explain (n=30) | 47 | [30,64] | 32 | [20,47] | 38 | [28,49] |
| Have heard of (n=21) | 19 | [9,36] | 32 | [20,47] | 27 | [18,38] |
| Not familiar (n=28) | 34 | [20,52] | 36 | [24,51] | 35 | [26,47] |
| **Total (n=79)** | 100 |  | 100 |  | 100 |  |

† Indirect oversight = non-practitioner that provides 1000 sows worth or less with direct veterinary services.

†† Direct oversight = practitioner or a non-practitioner that provides >1000 sows worth with direct veterinary services.

**Table 5**. **(Q12)** Self -reported level of confidence to assess aspects of a research paper, by role (direct vs. indirect).

|  | **Indirect Oversight†** | | **Direct Oversight††** | | **Overall** | |
| --- | --- | --- | --- | --- | --- | --- |
|  | Column % | 95% CI | Column % | 95% CI | Column % | 95% CI |
| **Study design used** |  |  |  |  |  |  |
| Complete confidence (n=26) | 38 | [22,56] | 30 | [19,45] | 33 | [24,45] |
| Some confidence (n=44) | 56 | [39,72] | 57 | [42,70] | 56 | [45,67] |
| No confidence (n=6) | 3 | [0,20] | 11 | [5,24] | 8 | [3,16] |
| Do not evaluate (n=2) | 3 | [0,20] | 2 | [0,14] | 3 | [1,10] |
| Total (n=78) | 100 |  | 100 |  | 100 |  |
| **Statistical methods used** |  |  |  |  |  |  |
| Complete confidence (n=14) | 19 | [9,36] | 17 | [9,31] | 18 | [11,28] |
| Some confidence (n=39) | 59 | [42,75] | 43 | [30,58] | 50 | [39,61] |
| No confidence (n=21) | 16 | [7,33] | 35 | [22,50] | 27 | [18,38] |
| Do not evaluate (n=4) | 6 | [2,22] | 4 | [1,16] | 5 | [2,13] |
| Total (n=78) | 100 |  | 100 |  | 100 |  |
| **Statistical Interpretation of results** | |  |  |  |  |  |
| Complete confidence (n=16) | 19 | [9,36] | 22 | [12,36] | 21 | [13,31] |
| Some confidence (n=42) | 59 | [42,75] | 50 | [36,64] | 54 | [43,65] |
| No confidence (n=16) | 16 | [7,33] | 24 | [14,39] | 21 | [13,31] |
| Do not evaluate (n=4) | 6 | [2,22] | 4 | [1,16] | 5 | [2,13] |
| Total (n=78) | 100 |  | 100 |  | 100 |  |

† Indirect oversight = non-practitioner that provides 1000 sows worth or less with direct veterinary services.

†† Direct oversight = practitioner or a non-practitioner that provides >1000 sows worth with direct veterinary services..

**Table 6A**. **(Q12)** Self -reported level of confidence to assess aspects of a research paper, by role (direct vs. indirect) with responses dichotomized* as ‘Confident’ or ‘No Confidence/Do not evaluate’.

|  | **Indirect Oversight†** | | **Direct Oversight††** | | **Overall** | |
| --- | --- | --- | --- | --- | --- | --- |
|  | Column % | 95% CI | Column % | 95% CI | Column % | 95% CI |
| **Study design used*** |  |  |  |  |  |  |
| Confident (n=70) | 94 | [78,98] | 87 | [73,94] | 90 | [81,95] |
| No confidence/ do not evaluate (n=8) | 6 | [2,22] | 13 | [6,27] | 10 | [5,19] |
| **Statistical methods used*** |  |  |  |  |  |  |
| Confident (n=53) | 78 | [60,89] | 61 | [46,74] | 68 | [57,78] |
| No confidence/ do not evaluate (n=25) | 22 | [11,40] | 39 | [26,54] | 32 | [22,43] |
| **Statistical Interpretation of results*** |  |  |  |  |  |  |
| Confident (n=58) | 78 | [60,89] | 72 | [57,83] | 74 | [63,83] |
| No confidence/ do not evaluate (n=20) | 22 | [11,40] | 28 | [17,43] | 26 | [17,37] |
| total (n=78) | 100 |  | 100 |  | 100 |  |

* Multiple response options for questions grouped into dichotomous options (See Table 1 in Additional File 3)

† Indirect oversight = non-practitioner that provides 1000 sows worth or less with direct veterinary services.

†† Direct oversight = practitioner or a non-practitioner that provides >1000 sows worth with direct veterinary services.

**Table 6B**. **(Q12)** Association of having confidence* with usually reading* versus not usually reading the methods section of journal articles

| Technical aspect | Prevalence ratio | 95% CI | P value |
| --- | --- | --- | --- |
| Confidence to assess study design | 1.8 | 0.5-6.2 | 0.46 |
| Confidence to assess statistical methods used | 1.5 | 0.8-2.8 | 0.23 |
| Confidence to assess study author interpretation of statistical results | 2.0 | 0.9-4.4 | 0.07 |

* dichotomised values

**Table 7**. **(Q4)** Ranking of first (1st), second (2nd), and third (3rd) choices for getting more information for difficult clinical cases, by role (direct vs. indirect).

|  | **Indirect Oversight†** | | **Direct Oversight††** | | **Overall** | |
| --- | --- | --- | --- | --- | --- | --- |
|  | Column% | 95% CI | Column% | 95% CI | Column% | 95% CI |
| **First (1st) choice** |  |  |  |  |  |  |
| Colleague (n=38) | 35 | [21,54] | 59 | [44,72] | 49 | [38,61] |
| Scientific journals (n=12) | 29 | [16,47] | 7 | [2,19] | 16 | [9,26] |
| Specialist (n=7) | 10 | [3,27] | 9 | [3,21] | 9 | [4,18] |
| General internet search (n=6) | 10 | [3,27] | 7 | [2,19] | 8 | [3,17] |
| Textbook (n=5) | 6 | [2,23] | 7 | [2,19] | 6 | [3,15] |
| AASV-L-digest (n=5) | 6 | [2,23] | 7 | [2,19] | 6 | [3,15] |
| Conference proceedings (n=3) | 3 | [0,20] | 4 | [1,16] | 4 | [1,12] |
| Veterinary websites or blogs (n=1) | 0 |  | 2 | [0,14] | 1 | [0,9] |
| **Total (n=77)** | 100 |  | 100 |  | 100 |  |
| **Second (2nd) choice** |  |  |  |  |  |  |
| Scientific journals (n=20) | 23 | [11,42] | 28 | [17,43] | 26 | [17,38] |
| Specialist (n=16) | 20 | [9,38] | 22 | [12,36] | 21 | [13,32] |
| General internet search (n=12) | 27 | [14,45] | 9 | [3,21] | 16 | [9,26] |
| Conference proceedings (n=9) | 3 | [0,21] | 17 | [9,31] | 12 | [6,21] |
| Colleague (n=5) | 7 | [2,24] | 7 | [2,19] | 7 | [3,15] |
| Textbook (n=5) | 7 | [2,24] | 7 | [2,19] | 7 | [3,15] |
| AASV-L-digest (n=4) | 7 | [2,24] | 4 | [1,16] | 5 | [2,13] |
| Veterinary websites or blogs (n=3) | 3 | [0,21] | 4 | [1,16] | 4 | [1,12] |
| Conference attendance (n=2) | 3 | [0,21] | 2 | [0,14] | 3 | [1,10] |
| **Total (n=76)** | 100 |  | 100 |  | 100 |  |
| **Third (3rd) choice** |  |  |  |  |  |  |
| Conference proceedings (n=13) | 14 | [5,32] | 20 | [10,34] | 17 | [10,28] |
| Scientific journals (n=11) | 14 | [5,32] | 15 | [7,29] | 15 | [8,25] |
| Specialist (n=9) | 21 | [9,40] | 7 | [2,19] | 12 | [6,22] |
| Colleague (n=9) | 17 | [7,36] | 9 | [3,21] | 12 | [6,22] |
| Textbook (n=9) | 14 | [5,32] | 11 | [5,24] | 12 | [6,22] |
| General internet search (n=9) | 10 | [3,28] | 13 | [6,27] | 12 | [6,22] |
| AASV-L-digest (n=8) | 3 | [0,22] | 15 | [7,29] | 11 | [5,20] |
| Conference attendance (n=3) | 3 | [0,22] | 4 | [1,16] | 4 | [1,12] |
| Veterinary websites or blogs (n=3) | 3 | [0,22] | 4 | [1,16] | 4 | [1,12] |
| Veterinary organization manuals (n=1) | 14 | [5,32] | 20 | [10,34] | 1 | [0,9] |
| **Total (n=75)** | 100 |  | 100 |  | 100 |  |

† Indirect oversight = non-practitioner that provides 1000 sows worth or less with direct veterinary services.

†† Direct oversight = practitioner or a non-practitioner that provides >1000 sows worth with direct veterinary services.

**Table 8**. **(Q11)** Self-reported frequency of reading sections of scientific journal articles, by role (direct vs. indirect)

|  | **Indirect Oversight†** | | **Direct Oversight††** | | **Overall** | |
| --- | --- | --- | --- | --- | --- | --- |
|  | % | 95% CI | % | 95% CI | % | 95% CI |
| **Abstract** |  |  |  |  |  |  |
| Usually(n=75) | 100 |  | 96 | [83,99] | 97 | [90,99] |
| Occasionally (n=2) | 0 |  | 4 | [1,17] | 3 | [1,10] |
| **Introduction** |  |  |  |  |  |  |
| Usually(n=45) | 59 | [42,75] | 55 | [41,69] | 57 | [46,68] |
| Occasionally (n=31) | 41 | [25,58] | 38 | [25,53] | 39 | [29,51] |
| Rarely or not at all (n=3) | 0 |  | 6 | [2,18] | 4 | [1,11] |
| **Materials and Methods** |  |  |  |  |  |  |
| Usually(n=33) | 41 | [25,58] | 43 | [30,58] | 42 | [32,54] |
| Occasionally (n=39) | 50 | [33,67] | 50 | [36,64] | 50 | [39,61] |
| Rarely or not at all (n=296) | 9 | [3,26] | 7 | [2,19] | 8 | [3,16] |
| **Results** |  |  |  |  |  |  |
| Usually(n=57) | 68 | [49,82] | 77 | [62,87] | 73 | [62,82] |
| Occasionally (n=21) | 32 | [18,51] | 23 | [13,38] | 27 | [18,38] |
| **Discussion** |  |  |  |  |  |  |
| Usually(n=55) | 66 | [48,80] | 72 | [58,83] | 70 | [58,79] |
| Occasionally (n=23) | 31 | [17,49] | 28 | [17,42] | 29 | [20,40] |
| Rarely or not at all (n=1) | 3 | [0,20] | 0 |  | 1 | [0,9] |
| **Conclusion** |  |  |  |  |  |  |
| Usually(n=66) | 81 | [64,91] | 89 | [76,95] | 86 | [76,92] |
| Occasionally (n=11) | 19 | [9,36] | 11 | [5,24] | 14 | [8,24] |
| **Conflict of Interest** |  |  |  |  |  |  |
| Usually(n=20) | 19 | [9,36] | 30 | [18,45] | 25 | [17,36] |
| Occasionally (n=32) | 44 | [28,61] | 38 | [25,53] | 41 | [30,52] |
| Rarely or not at all (n=27) | 38 | [22,55] | 32 | [20,47] | 34 | [24,46] |
| **Acknowledgements** |  |  |  |  |  |  |
| Usually(n=10) | 9 | [3,26] | 15 | [7,29] | 13 | [7,22] |
| Occasionally (n=26) | 28 | [15,46] | 37 | [24,52] | 33 | [24,45] |
| Rarely or not at all (n=42) | 63 | [44,78] | 48 | [34,62] | 54 | [43,65] |
| **Total (n=79)** | 100 |  | 100 |  | 100 |  |

† Indirect oversight = non-practitioner that provides 1000 sows worth or less with direct veterinary services.

†† Direct oversight = practitioner or a non-practitioner that provides >1000 sows worth with direct veterinary services.

**Table 9**. Self-reported scientific journal access **(Q15)** and article service awareness **(Q17)** by frequency of blocked access **(Q16)** (Often, Occasionally, Rarely, Not at all)

|  | **Often (n=18)** | | **Occasionally (n=26)** | | **Rarely (n=29)** | | **Not at all (n=6)** | | **Total** | |
| --- | --- | --- | --- | --- | --- | --- | --- | --- | --- | --- |
|  | % | 95% CI | Column % | 95% CI | % | 95% CI | % | 95% CI | % | 95% CI |
| **Reported number of fully accessible scientific journals through personal or work subscriptions** | | | | | | | | | | |
| 0 (n=3) | 6 | [1,32] | 4 | [0,23] | 3 | [0,22] | 0 |  | 4 | [1,11] |
| 1-2 (n=29) | 39 | [19,63] | 48 | [30,67] | 31 | [17,50] | 0 |  | 36 | [26,48] |
| 3-4 (n=12) | 17 | [5,42] | 19 | [8,38] | 7 | [2,24] | 33 | [8,74] | 15 | [9,25] |
| 5-6 (n=4) | 11 | [3,36] | 4 | [0,23] | 3 | [0,22] | 0 |  | 5 | [2,13] |
| >6 (n=32) | 28 | [12,52] | 26 | [13,46] | 55 | [37,72] | 67 | [26,92] | 40 | [30,51] |
| **Total (n=80)** | 100 |  | 100 |  | 100 |  | 100 |  | 100 |  |
| **Reported number of fully accessible scientific journals with outcomes dichotomized* to 0-6 or >6** | | | | | | | | | | |
| 0-6 (n=48) | 72 | [48,88] | 74 | [54,87] | 45 | [28,63] | 33 | [8,74] | 60 | [49,70] |
| >6 (n=32) | 28 | [12,52] | 26 | [13,46] | 55 | [37,72] | 67 | [26,92] | 40 | [30,51] |
| **Total (n=80)** | 100 |  | 100 |  | 100 |  | 100 |  | 100 |  |
| **Awareness of AASV journal article retrieval service - Get it For Me** | | | | | | | | | | |
| Yes (n=40) | 61 | [37,81] | 41 | [24,60] | 52 | [34,69] | 50 | [16,84] | 50 | [39,61] |
| No (n=40) | 39 | [19,63] | 59 | [40,76] | 48 | [31,66] | 50 | [16,84] | 50 | [39,61] |
| **Total (n=80)** | 100 |  | 100 |  | 100 |  | 100 |  | 100 |  |

* Multiple response options for question grouped into dichotomous option (See Table 2 in Additional File 3)

**Table 10**. **(Q18)** Association of selection of a preferred reading material format (for keeping current with infectious disease topics) with veterinarian role (direct versus indirect).

| Reading material format – selected options | Prevalence ratio (PR) | 95% CI | P value (2-sided Fisher’s Exact) |
| --- | --- | --- | --- |
| Narrative review of body of evidence | 0.99 | 0.82 - 1.20 | 1.000 |
| 3 page summary of body of evidence | 0.98 | 0.76- 1.26 | 1.000 |
| 1 page summary of body of evidence | 1.14 | 0.85- 1.51 | 0.46 |
| Individual primary research paper | 0.90 | 0.59-1.39 | 0.65 |
| 1 page summary of individual primary research paper | 1.34 | 0.89-2.00 | 0.25 |
| Other | 0.93 | 0.82-1.04 | 0.30 |

**Table 11A**. **(Q18)** Respondent self-reported time***(Q7)**, skill***(Q10,12)** and access***(Q11)** by their choice of individual primary research papers (IPRP) as a preferred reading format for keeping current with a specific disease topic (IPRP not selected vs IPRP selected)

|  | **IPRP not selected†** | | **IPRP selected††** | | **Overall** | | |
| --- | --- | --- | --- | --- | --- | --- | --- |
|  | % | 95% CI | % | 95% CI | % | 95% CI | |
| Q7: Time spent per week keeping current with infectious disease research | | | | | |  |  |
| > 1 hour (n=37) | 33 | [21,49] | 62 | [45,76] | 47 | [36,58] | |
| 1 hour or less (n=42) | 67 | [51,79] | 38 | [24,55] | 53 | [42,64] | |
| Total (n=79) | 100 |  | 100 |  | 100 |  | |
| Q10: Skill - Familiarity with epidemiologic term 'confounding bias' | | | | | |  |  |
| Can explain (n=38) | 40 | [26,55] | 57 | [40,72] | 48 | [37,59] | |
| Cannot explain (n=42) | 60 | [45,74] | 43 | [28,60] | 53 | [41,63] | |
| Total (n=80) | 100 |  | 100 |  | 100 |  | |
| Q11: Access - Frequency of reading materials and methods section of published primary research studies | | | | | | |  |
| Usually read (n=34) | 36 | [23,51] | 51 | [35,67] | 43 | [32,54] | |
| Not usually read (n=45) | 64 | [49,77] | 49 | [33,65] | 57 | [46,68] | |
| Total (n=79) | 100 |  | 100 |  | 100 |  | |
| Q12: Skill - Level of confidence to assess appropriateness of statistical methods used in a primary research study | | | | | | | |
| Confident (n=54) | 52 | [37,67] | 86 | [71,94] | 68 | [57,78] | |
| No confidence/do not evaluate (n=25) | 48 | [33,63] | 14 | [6,29] | 32 | [22,43] | |
| Total (n=79) | 100 |  | 100 |  | 100 |  | |

†IPRP not selected = Individual primary research paper was not selected as a preferred reading material format for keeping current

††IPRP selected = Individual primary research paper was selected as a preferred reading material format for keeping current

* Multiple response options for questions grouped into dichotomous options (See Table 2 in Additional File 3)

**Table 11B**. **(Q18)** Association of time, skill and access for respondents with not selecting IPRP* versus selecting IPRP as a preferred reading format

| Self-reported time, skill and access (dichotomous response options**) | Prevalence ratio | 95% CI | P value |
| --- | --- | --- | --- |
| Q7: Time - Spend an hour or less per week keeping current with infectious disease research | 1.8 | 1.1-2.8 | 0.01 |
| Q10: Skill-Cannot explain the epidemiologic term ‘confounding bias’ | 1.4 | 0.9-2.2 | 0.18 |
| Q11: Access-Do not usually read materials & methods section of published individual primary research papers | 1.4 | 0.9-2.0 | 0.16 |
| Q12: Skill-No confidence or do not evaluate the appropriateness of the statistical methods used in a research study | 3.5 | 1.5-8.5 | 0.001 |

* IPRP = Individual Primary peer-reviewed Research Paper

** Multiple response options for questions grouped into dichotomous options (See Table 2 in Additional File 3)

**Table 12**. **(Q18)** Association of selected combinations of choices* of reading material format options with direct^†^ veterinary role versus indirect^††^ veterinary role

| Reading format - selected combination of 2 options | Prevalence ratio (PR) | 95% CI | P value |
| --- | --- | --- | --- |
| Both of body of evidence and individual primary study format options | 1.03 | 0.71-1.51 | 1.000 |
| Individual primary study format options as a single choice only, or as both options | 1.08 | 0.78-1.48 | 0.81 |
| Body of evidence format options as a single choice only or as both options | 1.00 | 0.81-1.24 | 1.000 |
| Other options selected as a single choice only or in combination with other formats | 0.93 | 0.82-1.04 | 0.30 |

*Respondent could choose up to two options.

† Direct oversight = practitioner or a non-practitioner that provides >1000 sows worth with direct veterinary services.

†† Indirect oversight = non-practitioner that provides 1000 sows worth or less with direct veterinary services.
